# Do managed bees have negative effects on wild bees?: A systematic review of the literature

**DOI:** 10.1371/journal.pone.0189268

**Published:** 2017-12-08

**Authors:** Rachel E. Mallinger, Hannah R. Gaines-Day, Claudio Gratton

**Affiliations:** Department of Entomology, University of Wisconsin-Madison, Madison, Wisconsin, United States of America; University of Guelph, CANADA

## Abstract

Managed bees are critical for crop pollination worldwide. As the demand for pollinator-dependent crops increases, so does the use of managed bees. Concern has arisen that managed bees may have unintended negative impacts on native wild bees, which are important pollinators in both agricultural and natural ecosystems. The goal of this study was to synthesize the literature documenting the effects of managed honey bees and bumble bees on wild bees in three areas: (1) competition for floral and nesting resources, (2) indirect effects via changes in plant communities, including the spread of exotic plants and decline of native plants, and (3) transmission of pathogens. The majority of reviewed studies reported negative effects of managed bees, but trends differed across topical areas. Of studies examining competition, results were highly variable with 53% reporting negative effects on wild bees, while 28% reported no effects and 19% reported mixed effects (varying with the bee species or variables examined). Equal numbers of studies examining plant communities reported positive (36%) and negative (36%) effects, with the remainder reporting no or mixed effects. Finally, the majority of studies on pathogen transmission (70%) reported potential negative effects of managed bees on wild bees. However, most studies across all topical areas documented the potential for impact (e.g. reporting the occurrence of competition or pathogens), but did not measure direct effects on wild bee fitness, abundance, or diversity. Furthermore, we found that results varied depending on whether managed bees were in their native or non-native range; managed bees within their native range had lesser competitive effects, but potentially greater effects on wild bees via pathogen transmission. We conclude that while this field has expanded considerably in recent decades, additional research measuring direct, long-term, and population-level effects of managed bees is needed to understand their potential impact on wild bees.

## Introduction

The status of bees worldwide is currently a topic of research and conservation concern [1–5]. There are approximately 20,000 species of bees worldwide, and these insects are arguably the most important pollinators for both crop and wild plants [6–8]. Numerous factors may be threatening bees including habitat loss and fragmentation, pesticides, and disease [3, 9–10]. In addition, the increasingly widespread use of managed bees may have negative effects on wild bee populations (reviewed by [11, 12]). Managed bees, including honey bees, bumble bees, and some solitary bees, have become an integral component of agriculture due to a rising demand for pollinator-dependent crops (e.g., almonds, tree fruits, berries), and without which many farms would likely experience pollination deficits [13–14]. However, the use of managed bees may negatively affect wild bee abundance or diversity, which could in turn impact food production since a diverse wild bee community has been found to increase pollination rates and subsequent crop yields even when managed bees are present [15–19]. Furthermore, in natural habitats, a diverse wild bee community is integral for maintaining plant diversity and ecosystem function [20–21]. Thus, identifying and quantifying the factors that affect wild bees is essential for bee conservation and to ensure pollination services within both managed and natural habitats.

**Fig 1:**
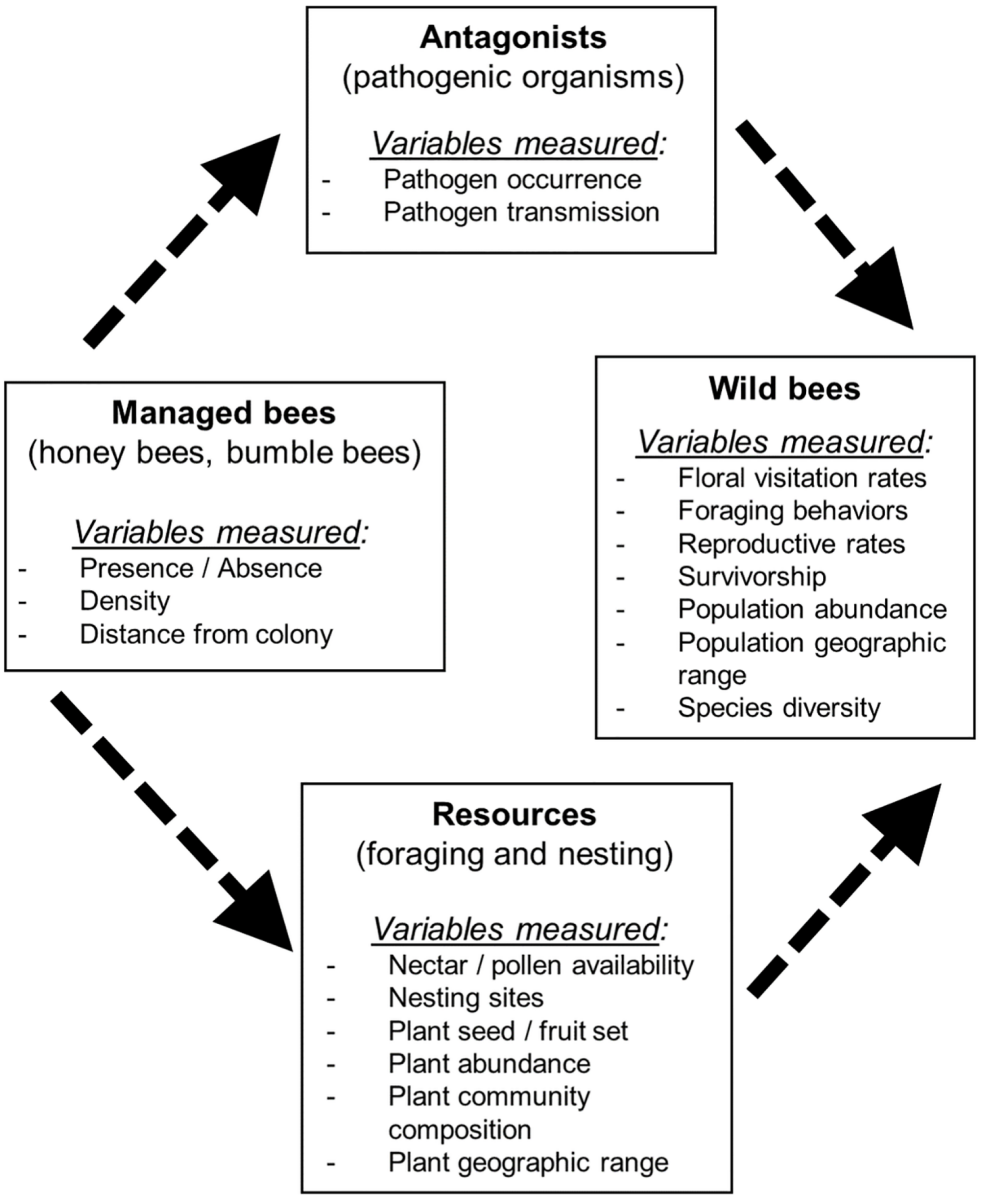
Hypothesized interactions between managed bees and wild bees. Wild and managed bees may interact indirectly (dashed lines) through either bottom-up effects on shared resources (including pollen, nectar, and nesting sites), or by altering top-down interactions through shared antagonists such as pathogenic organisms.

There are several ways in which managed bees could affect wild bees including through competition over finite resources such as nectar, pollen, or nesting habitat (Fig 1). Competition with managed bees for pollen and nectar may induce changes in wild bee floral use and niche breadth, with potential consequences for bee fitness. While the majority of wild bees are polylectic and potentially able to modify foraging behaviors in the presence of honey bees, competition could still have negative effects if wild bees are forced to forage on less nutritious plants, spend more time searching for flowers that are unoccupied or whose resources have not yet been depleted, or forage further from their nests [22–26]. Additionally, in regions where managed bees escape and establish in the wild, they could compete with wild bees for nesting sites such as tree or ground cavities [27]. The extent of competitive effects, however, could depend on many factors including overall resource availability, the degree of niche overlap between managed and wild bee species, and densities of both managed and wild bees.

Managed bees could also affect resource availability for wild bees by changing plant community composition. Previous studies have shown that in some regions where managed bees are exotic, they preferentially forage on exotic plants [28–30]. These foraging preferences can form invasive mutualisms whereby exotic pollinators and plants facilitate each other’s spread in non-native regions, subsequently reducing populations of native plants [29]. The decline of native plants could then disrupt plant-pollinator networks, potentially leading to a loss of native bee species [1, 31]. However, while some bee species are specialists and may therefore be sensitive to the loss of native plants, the majority of wild bees are generalists and may therefore be resilient to changes in plant community composition [32–34].

Finally, managed bees may affect wild bees through shared antagonists, specifically pathogenic organisms. Most managed bees, including honey bees and bumble bees, are social species and occur in high densities, potentially making them more likely to harbor pathogens than their solitary wild counterparts [35–36]. The movement of these managed bees across large regions for crop pollination could increase their potential to spread such pathogens to wild bees. Furthermore, managed bees are often deployed outside of their native ranges, and can thus introduce novel, invasive pathogens [11, 28, 37]. Transmission of infectious agents by managed bees to wild bees can occur via contaminated pollen [38], feces [39], or through contact on shared foraging resources [40]. Shared pathogens have been found between managed and wild bees of the same species, closely related species, and distantly related species, suggesting that transmission of antagonists is possible and has the potential to affect a broad wild bee community [41–46]. The extent to which managed bees transmit pathogens to wild bees, and the effects of such antagonists on wild bee fitness, is likely to vary with the density and health of managed bees as well as the type of pathogen.

Two previous review papers by Goulson [11] and Paini [12] on this general topic found much circumstantial evidence for competition between managed and wild bees, but very little evidence that such competition has population-level or long-term effects on wild bees. Additionally, Goulson [11] concluded that exotic managed bees negatively affect plant community composition through the pollination of invasive exotic weeds, but the effects of native managed bees on plant communities were not addressed [11]. Furthermore, the effect of managed bees on wild bees via the transmission of natural enemies, including pathogens and parasites, was not well-covered in either review because there were few studies to date on that topic. Since the publication of these reviews in 2003 and 2004, no systematic review has been conducted on the overall effects of managed bees on wild bees. And with the increasing use of managed bees to meet agricultural demand [13], the effects of managed bees on wild flora and fauna is a mounting issue. Managed bees may be necessary in agricultural landscapes as crop pollinators, and may also benefit from supplemental foraging in natural habitats. Thus, this topic is relevant not only for growers, beekeepers, and the commercial bee industry, but for public land managers who may be considering the placement of managed bees within conservation areas or other public lands.

In this paper, we synthesize the literature on the effects of managed bees, here restricted to honey bees *Apis* spp. and bumble bees *Bombus* spp., on wild bees. Though there are other species of managed bees, honey bees and bumble bees are the most commonly used worldwide and relatively well researched. We searched for and synthesized papers that fell into three broad topical areas by which managed bees can affect wild bees: 1) competition for shared resources; 2) changes in plant community composition, specifically an increase in exotic plants and a subsequent decline in native plants, which is both a conservation concern in itself and has the potential to negatively affect native wild bees, and 3) the transmission of shared pathogens. While there may be other pathways by which managed bees affect wild bees, such as interspecific mating [47], these three topical areas are relatively well-studied and encompass those covered in earlier reviews [11–12]. Our findings have implications for the management of pollinators in natural and agricultural systems and for the conservation of wild bees.

## Materials and methods

We performed a systematic search of the literature using Web of Knowledge/Web of Science (ISI Thompson-Reuters, webofknowledge.com) to identify studies that examined the effects of managed bees on wild bees via competition, changes in plant communities, and transmission of pathogens. Due to the broad nature of our focal question, we synthesized the literature with a systematic review as opposed to a meta-analysis. In addition, the studies in our review measured different metrics associated with both managed bees and wild bees (e.g., bee visitation rates, abundance, diversity, reproductive rates) that would have been difficult to standardize in a meta-analysis (Fig 1). Instead, as part of our systematic review, we used a vote-counting analysis to quantify the variables measured, and results found, across studies. We focused our review on the most common and widely used managed bees, honey bees and bumble bees. The use of other managed bees, including the orchard mason bee *Osmia lignaria* Say and alfalfa leafcutter bee *Megachile rotundata* Fabricius, is more limited to specific crops and geographic regions, resulting in fewer studies on these bees, and thus we excluded them from this systematic review.

To search for the effects of managed honey bees on wild bees via competition, changes in plant communities, and transmission of pathogens, including pathogenic parasites, we used the search terms: (“Apis mellifera” OR “honey bee” OR honeybee) AND (competition OR disease OR pathogen OR (pollin* AND (exotic OR invasive))). To identify studies that examined the effects of managed bumble bees, we used the search terms: (Bombus OR “bumble bee” OR bumblebee) AND (competition OR disease OR pathogen OR (pollin* AND (exotic OR invasive))). We additionally conducted a more general search to find studies that were not identified by the previous searches using the terms: “managed bee” AND (competition OR disease OR pathogen OR (pollin* AND (exotic OR invasive))). We included all papers returned by these searches beginning in 1900 and through the end of 2016. We additionally reviewed all articles that were cited by the two older non-systematic reviews on this topic [11–12], and searched for all recent articles that cited these two reviews [11–12].

We evaluated every article returned by our searches for whether or not it broadly addressed one of our three topical areas: competition between managed and wild bees, effects of managed bees on plant communities (natives vs. exotics), and transmission of pathogens, including pathogenic parasites, from managed to wild bees. Studies that did not broadly fall into the three topical areas, as well as review papers, were excluded. Additionally, we excluded papers that were not peer-reviewed (e.g. theses, conference proceedings) and papers not available in English. Furthermore, to be included in our review, studies needed to measure some response metric of either wild bees or plants (dependent variables, e.g., foraging behavior, abundance, reproductive rates) and relate that to a measured or assumed aspect of managed bee “intensity” (independent variable, e.g., presence/absence, before/after introduction, distance from colony, abundance). A study measuring pathogen presence in only managed bees, for example, would not be included if it did not also measure a wild bee response, regardless of any implications for wild bees discussed within the paper. For all studies, we recorded which topical area was addressed, the managed bee species examined and whether it was native to the study region, the wild bee taxa examined, the location and context of the study (e.g. field vs. lab), the independent managed bee variables measured, the dependent response variables measured (i.e. wild bee or plant metrics), and any additional explanatory or mechanism variables measured. We found a variety of independent and dependent variables across studies, and we did not discriminate among these variables for inclusion in this study. Furthermore, while we noted mechanistic or explanatory variables, studies did not need to measure such variables for inclusion in the study.

We additionally scored each article for whether the authors reported negative, positive, mixed, or no effects of managed bees. Consistent across all three topical areas, scores are from the perspective of native wild bees or native plants, where a negative score means that some measure of their performance decreases with managed bees, and a positive score means that performance improves with managed bees. Specifically, for competitive effects of managed bees on wild bees, “negative” (-) means that managed bees compete with wild bees and/or increased intra- or interspecific competition among wild bees, “no effect” (0) means that managed bees did not compete with wild bees and/or had no competitive effect on wild bees, and "mixed effects" means that responses varied across different wild bee species or different measures of competition. While we did not specifically search for studies examining mutualism or commensalism, a “positive” effect (+) in this area would include studies examining potential competitive effects but finding positive relationships between managed and wild bees (e.g. a positive correlation between abundances or visitation rates of managed and wild bees).

For the effects of managed bees on plant communities, “negative” (-) means that managed bees had a negative effect on native plants (e.g., decreased plant abundance) and/or a positive effect on exotic plants (e.g., increased plant abundance), “positive” (+) means that managed bees had a positive effect on native plants and/or a negative effect on exotic plants, “no effect” (0) means that managed bees had no effect on plant communities, and "mixed effects" means that responses varied by plant species or across different plant variables measured. Increases in native plants and/or decreases in exotic plants was considered to be a positive response because restoring native plant communities, a common bee conservation goal, is often associated with increases in native wild bees [48–49].

For evaluating the potential effects of managed bees on wild bees via pathogens, "negative” (-) means that managed bees increased pathogen occurrence in wild bees or that managed bee pathogens had a negative effect on wild bees including on fitness, abundance, diversity, etc., “no effect” (0) means that managed bees had no effect on the occurrence of pathogens in wild bees, or that managed bee pathogens had no effect on wild bees, and “mixed effects" means that effects varied across wild bee species, pathogens, or response variables examined. As it is unlikely that managed bees could have a positive effect on wild bees in this area (e.g. decrease pathogen occurrence), and pathogens by definition do not have a positive effect on their host, there were no positive effects found in this category.

## Results

**Fig 2:**
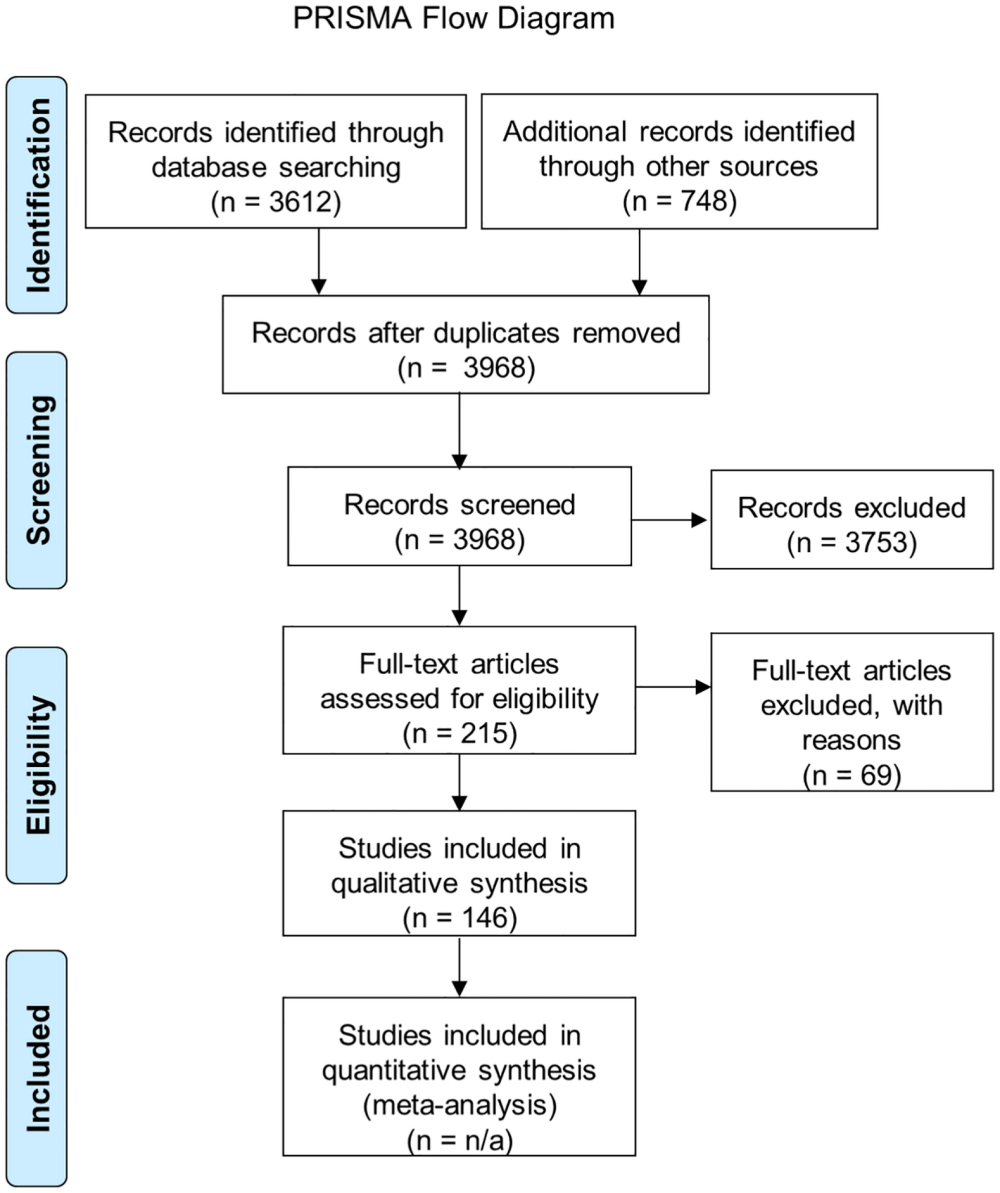
PRISMA flow diagram. A flow diagram showing the process for a systematic review including the number of studies processed, reviewed, and analyzed at each step in the review process.

Our search of the literature identified 146 studies that fit our inclusion criteria and broadly addressed the effects of managed bees on wild bees via competition, changes in plant communities (specifically changes in exotic and native plant populations), or transmission of pathogens (Fig 2, S1 References). Of these studies, 72 addressed competition, 41 addressed plant communities, 6 studied both competition and plant communities, and 27 addressed pathogens. The majority of studies examining competition and plant communities focused on managed honey bees *Apis* spp. (number of studies, n = 59 and 36, respectively) with fewer studies on managed bumble bees (n = 17 and 6, respectively) or on both (n = 2 and 5, respectively) (Tables 1 and 2). However, studies on pathogens were more evenly split between those studying managed honey bees (n = 15) and managed bumble bees (n = 10) (Table 3).

**Table 1:** Studies published from 1900–2016 examining potential competitive effects of managed bees on wild bees. For all studies, we recorded the species of managed and wild bees, and indicated whether managed bees were native or exotic to the study region, the location (continent and country) and context of the study including field (natural, semi-natural, developed, agricultural, or experimental plot), lab, or greenhouse, and all variables measured, including the managed bee metric (independent variable), wild bee metric (dependent variable), and any explanatory or mechanistic variables. The overall competitive effect of managed bees on wild bees, as reported by the study, is also recorded and noted as positive (+), neutral (0), negative (-), or mixed.

Reference	Managed bee species (* indicates exotic range)	Wild bee species	Location	Context	Managed bee metric (independent variable)	Explanatory mechanism variable	Wild bee metric (dependent variable)	Reported effect
Abe et al. 2010	Apis mellifera*	*Xylocopa Ogasawarensis* & endemic small bees	Asia (Japan)	field (natural)	honey bee presence/absence and/or abundance	none	distribution	0
Aizen & Feinsinger 1994	*A*. mellifera*	many	South America (Argentina)	field (natural)	visitation rates	none (different responses to forest fragmentation speculated)	visitation rates	-
Aizen et al. 2011	Bombus ruder-atus*	*Bombus dahlbomii*	South America (Argentina)	field (natural)	foraging behavior (floral preferences, nectar removal), visitation rates	nectar availability	foraging behavior (floral preferences, nectar removal), visitation rates	0
Badano & Vergara 2011	*A*.mellifera*	many	North America (Mexico)	field (agricultural)	abundance	none	diversity	-
Balfour et al. 2013	*A*.*mellifera*	*Bombus terrestris/lucorum*, *Bombus pascuorum*, *Bombus lapidarius*	Europe (UK)	field (experiment plots)	visitation rates, foraging behavior (handling times, number of floral probes)	tongue length	visitation rates, foraging behavior (handling time, number of floral probes)	0
Balfour et al. 2015	*A*.*mellifera*	*Bombus* spp.	Europe (UK)	field (experiment plots)	visitation rates, foraging behavior (search time, extraction time, etc.)	nectar volume & sugar concentration, energetic returns per flower	visitation rates, foraging behavior (search time, extraction time, etc.)	0
Batra 1999	*A*. mellifera*	many	North America (USA)	field (semi-natural)	visitation rates	none	visitation rates	0
Cane & Tepedino 2017	*A*. mellifera*	many (average-sized solitary bees)	North America (USA)	lab	amount of pollen collected per colony	none	amount of pollen needed to produce one offspring	-
Carneiro & Martins 2012	*A*. mellifera scutellata*	many	South America (Brazil)	field (natural)	visitation rates	pollen depletion	visitation rates	-
Connor & Neumeier 1995	*A*. mellifera*	many	North America (USA)	field (natural)	visitation rates	none	visitation rates	-
Dohzono et al. 2008	*B*. terrestris*	*Bombus ardens*, *Bombus hypocrita*	Asia (Japan)	field (natural)	presence/absence	nectar robbing & collection	visitation rates	-
Dupont et al. 2004	*A*. mellifera*	*Anthophora alluaudi*, *Eucera gracilipes*	Canary Islands	field (natural)	abundance	nectar depletion	visitation rates	-
El Shafie et al. 2002	Apis florea*	*A*. *mellifera sudanensis*	Africa (Sudan)	field (agricultural)	foraging behavior (types of pollen collected), visitation rates	none (niche partitioning implied)	foraging behavior (type of pollen collected), visitation rates	0
Elbgami et al. 2014	*A*. mellifera*	*B*. *terrestris* ^1^	Europe (UK)	field (agricultural)	distance from apiary	none	individual bee weight & reproductive success	-
Esterio et al. 2013	*B*. terrestris*	*B*. *dahlbomii*	South America (Chile)	field (natural)	visitation rates, foraging behavior (number of pollen grains carried & deposited)	none	visitation rates, foraging behavior (number of pollen grains carried & deposited)	0
Forup & Memmot 2005	*A*. *mellifera*	*Bombus* spp.	Europe (UK)	field (natural)	abundance, foraging behavior (diet breadth)	tongue length	abundance, diversity, foraging behavior (diet breadth)	-/0
Franco et al. 2009	*A*. mellifera*	*Bombus atratus*	South America (Brazil)	field (natural)	foraging behavior (plant use, diet breadth)	niche overlap	foraging behavior (plant use, diet breadth)	-/0
Ginsberg 1983	*A*. mellifera*	many	North America (USA)	field (semi-natural)	foraging behavior (plant preferences & foraging period)	niche overlap	foraging behavior (plant preferences & foraging period)	-/0
Goras et al. 2016	*A*. *mellifera*	many	Europe (Greece)	field (natural)	hive density	none	visitation rates, foraging behavior (visit duration)	0
Goulson & Sparrow 2009	*A*. *mellifera*	*B*. *pascuorum*, *B*. *lucorum*, *B*. *lapidarius*, *B*. *terrestris*	Europe (UK)	field (semi-natural)	presence/absence	none	thorax width	-
Goulson et al. 2002	*A*. mellifera*, Bombus terrestris*	many	Australia	field (natural, semi-natural, & developed)	presence/absence	niche overlap	abundance, diversity, & foraging behavior (floral preference)	-/0
Gross 2001	*A*. mellifera*	many	Australia	field (natural)	abundance, visitation rates	none	abundance, visitation rates	-
Gross & Mackay 1998	*A*. mellifera*	many	Australia	field (natural)	visitation rates	direct displacement interactions	visitation rates	-
Herbertsson et al 2016	*A*. *mellifera*	*Bombus* spp.	Europe (Sweden)	field (agricultural)	presence/absence	tongue length, thorax width	density	-/0
Hingston & McQuilan 1998	*B*. terrestris*	many	Australia	field (natural)	foraging behavior (diet breadth)	niche overlap	foraging behavior (diet breadth)	-
Hingston & McQuilan 1999	*B*. terrestris*	*Chalicodoma* spp.	Australia	field (natural)	presence/absence	none (nectar availability implied)	visitation rates, foraging behavior (foraging time)	-
Holmes 1964	*A*. mellifera*	*Bombus* spp.	North America (USA)	field (developed)	visitation rates	none	visitation rates	-
Horskins & Turner 1999	*A*. mellifera*	many	Australia	field (natural)	foraging behavior (temporal foraging patterns, stigma contact, nectar vs. pollen collecting trips)	nectar availability	foraging behavior (temporal foraging patterns, stigma contact, nectar vs. pollen collecting trips)	0
Hudewenz & Klein 2013	*A*. *mellifera*	many	Europe (Germany)	field (natural)	distance to hive, presence/absence	none	visitation rates, number of nests	-
Hudewenz & Klein 2015	*A*. *mellifera*	*Osmia bicornis*	Europe (Germany)	field (experiment plots)	abundance	interspecific displacement, visitation rates, niche breadth & overlap	number of nests & brood cells	-
Inari et al. 2005	*B*. terrestris*	*B*. *ardens*	Asia (Japan)	field (agricultural & semi-natural)	abundance, distance from greenhouse	none	abundance	-
Ings et al. 2006	*B*. terrestris dalmatinus*	*B*. *terrestris audax*	Europe (UK)	field (natural)	foraging behavior, visitation rates, production of new queens & males	none	foraging behavior, visitation rates, production of new queens & males	-
Inoue & Yokoyama 2010	*B*. terrestris*	*B*. *hypocrita sapporoensis*, *Bombus schrencki albidopleuralis*, *Bombus pseudobaicalensis*, *Bombus diversus tersatus*	Asia (Japan)	field (natural)	foraging behavior (diet breadth), reproductive capacity, temporal changes in abundance	niche overlap	temporal changes in abundance, foraging behavior (diet breadth)	-
Inoue et al. 2010	*B*. terrestris*	*Bombus ignitus*	Asia (Japan)	field (experiment plot)	foraging behavior (foraging load, foraging efficiency)	tongue length	foraging behavior (foraging load, foraging efficiency)	-
Ishii et al. 2008	*B*. terrestris*	*B*. *diversus tersatus*, *B*. *pseudobaicalensis*, *B*. *hypocrita sapporoensis*	Asia (Japan)	field (agricultural & natural)	habitat occupancy, foraging behavior (floral preferences)	flower morphology & tongue length	habitat occupancy, foraging behavior (floral preferences)	-
Kajobe 2007	*A*. *mellifera*	*Meliponula bocandei*, *Meliponula nebulata*	Africa (Uganda)	field (natural)	foraging behavior (diversity of pollen collected)	bee body & colony size	foraging behavior (diversity of pollen collected)	-/0
Kato & Kawakita 2004	*A*. mellifera*	many	New Caledonia	field (natural)	foraging behavior (plant use)	none	foraging behavior (plant use)	-
Kato et al. 1999	*A*. mellifera*	many	Bonin Islands	field (natural)	relative abundance	none	relative abundance	-
Kuhn et al. 2006	*A*. *mellifera*	*Megachile lapponica*	Europe (Germany)	field (natural)	density	none	visitation rates, foraging behavior (duration of foraging flights), brood cell construction	0
Lindstrom et al. 2016	*A*. *mellifera*	many	Europe (Sweden)	field (agricultural)	presence/absence, density	none	density	-
Lye et al. 2011	*B*. terrestris*	many	North America (USA)	field (agricultural)	presence/absence	none	visitation rates	0
Martins 2004	*A*. *mellifera*	many	Africa (Kenya)	field (natural)	visitation rates, foraging behavior (temporal foraging patterns, plant use)	direct displacement, nectar & pollen removal/depletio-n	visitation rates, foraging behavior (temporal foraging patterns, plant use)	-
Menezes et al. 2007	*A*. mellifera*	*Scaptotrigona* spp.	South America (Brazil)	field (experiment plot)	presence/absence	none (resource partitioning implied)	visitation rates, foraging behavior (floral preference)	-
Morales et al. 2013	Bombus ruderatus*, *B*. terrestris*	*B*. *dahlbomii*	South America (Chile)	field (natural)	temporal trends in regional abundance, geographic distribution	none	temporal trends in regional abundance, geographic distribution	-
Nagamitsu et al. 2007a	*B*. terrestris*	*B*. *ardens*, *B*. *hypocrita*	Asia (Japan)	field (experiment plot)	presence/absence	nectar availability	queen body mass, colony mass	0
Nagamitsu et al. 2007b	*B*. terrestris*	*B*. *hypocrita*, *B*. *ardens*, *B*. *diversus*	Asia (Japan)	field (natural)	abundance	tongue length	abundance, body size	0
Nagamitsu et al. 2010	*B*. terrestris*	*B*. *ardens*, *B*. *hypocrita*	Asia (Japan)	field (natural)	presence/absence	tongue length	abundance, worker body size	-
Nakamura 2014	*B*. terrestris*	*B*. *pseudobaicalensis*, *B*. *hypocrita sapporoensis*	Asia (Japan)	field (developed)	visitation rates, foraging behavior (pollen type & diversity on body)	niche overlap	visitation rates, foraging behavior (pollen type & diversity on body)	0/-
Neumayer 2006	*A*. *mellifera*	many	Europe (Austria)	field (natural)	distance from hive, presence/absence	nectar availability	visitation rates/local abundance	-
Nielsen et al. 2012	*A*. *mellifera*	many	Europe	field (natural)	visitation rates	none	visitation rates	-/0/+
Nishikawa & Shimamura 2015	*B*. terrestris*	*B*. *hypocrita*, *Bombus deuteronymus*	Asia (Japan)	field (natural)	visitation rates	tongue length, head width, niche overlap	visitation rates	0
Paini & Roberts 2005	*A*. mellifera*	*Hylaeus alcyoneus*	Australia	field (natural)	presence/absence	none	fecundity (number of nests, number of eggs per nest, progeny mass)	-
Paini et al. 2005	*A*. mellifera*	*Megachile* spp.	Australia	field (natural)	presence/absence	none (temperature adaptations implied)	reproductive success	0
Pedro & Camargo 1991	*A*. mellifera*	many	South America (Brazil)	field (semi-natural)	relative abundance, foraging behavior (floral preference)	none	relative abundance, foraging behavior (floral preference)	0
Pick & Schlindwein 2011	*A*. mellifera*	*Melitoma segmentaria*, *Melitoma osmioides*, *Melitomella murihir*, *Lithurgus huberi*	South America (Brazil)	field (natural)	foraging behavior (floral preferences), visitation rates	pollen removal	foraging behavior (floral preferences), visitation rates	0
Pinkus-Rendon et al. 2005	*A*. mellifera*	*Peponapis limitaris*, *Partamona bilineata*	North America (Mexico)	field (agricultural)	visitation rates, foraging behavior (plant use)	niche overlap, direct displacement interactions	visitation rates, foraging behavior (plant use)	-
Pleasants 1981	*A*. mellifera*	*Bombus* spp.	North America (USA)	field (experiment plots)	presence/absence	tongue length	abundance	-
Rogers et al. 2013	*A*. mellifera*	*Bombus impatiens*	North America (USA)	field (experiment plots)	response to intra & interspecific physical encounters at flowers	none	response to intra & interspecific physical encounters at flowers	-
Roubik 1978	*A*. mellifera*	many	South America (French Guiana)	field (natural)	presence/absence	none	flower visitation rates, foraging behavior (duration of floral visits)	-/0
Roubik 1980	*A*. mellifera*	*Melipona* spp., *Trigona* spp.	South America (French Guiana)	field (natural)	visitation rates to feeders	partitioning & displacement interactions at feeders	visitation rates to feeders	0/-
Roubik 1983	*A*. mellifera*	*Melipona favosa*, *Melipona fulva*	South America (French Guiana)	field (natural)	presence/absence, number of hives, amounts of brood, honey, & pollen in hive	none	amounts of brood, honey, & pollen in nest	0
Roubik et al. 1986	*A*. mellifera*	many	North America (Panama)	field (natural)	rate of forager return, foraging behavior (type, quantity, & quality of pollen & nectar gathered)	niche overlap	rate of forager return, foraging behavior (type, quantity & quality of pollen & nectar gathered)	-/0
Roubik & Villanueva-Gutierrez 2009	*A*. mellifera*	many	North America (Mexico)	field (natural)	presence/absence, foraging behaviors (plant use)	niche overlap	abundance, foraging behavior (pollen identity & diversity)	0
Roubik & Wolda 2001	*A*. mellifera*	many	North America (Panama)	field (natural)	presence/absence, abundance	none	abundance	0
Schaffer et al. 1979	*A*. mellifera*	*Bombus sonorous*, *Xylocopa arizonensis*	North America (USA)	field (natural)	visitation rates, foraging behavior (resource collection)	none	visitation rates, foraging behavior (resource collection)	-
Schaffer et al 1983	*A*. mellifera*	many	North America (USA)	field (natural)	presence/absence	nectar standing crop	visitation rates	-
Semida & Elbanna 2006	*A*. *mellifera*	many	Africa (Egypt)	field (natural)	visitation rates	none	visitation rates	-/0
Shavit et al. 2009	*A*. *mellifera*	many	Asia (Israel)	field (natural)	presence/absence	none	foraging behavior (temporal foraging patterns, plant use), visitation rates	-/0
Smith-Ramirez et al. 2014	*A*. mellifera*, *B*. terrestris*	many	South America (Chile)	field (natural)	visitation rates	none	visitation rates	-
Sugden & Pyke 1991	*A*. mellifera*	*Exoneura asimillima*	Australia	field (natural)	presence/absence	none	colony survival, developmental stage & sex ratios, relative frequency of founder vs. established colonies	-
Steffan-Dewenter & Tscharntke 2000	*A*. *mellifera*	many	Europe (Germany)	field (natural)	density, visitation rates	niche overlap	abundance, diversity, number of nests, number of brood cells, visitation rates	0
Tepedino et al. 2007	*A*. mellifera*	many	North America (USA)	field (agricultural)	visitation rates, distance from hive	none	visitation rates	0
Thomson 2004	*A*. mellifera*	*B*. *occidentalis*	North America (USA)	field (natural)	distance from hive	foraging effort devoted to pollen collection	foraging behavior (pollen vs. nectar collection, forager return rates), reproductive success	-
Thomson 2006	*A*. mellifera*	*Bombus* spp.	North America (USA)	field (natural)	foraging behavior (plant use), visitation rates, distance from hive	niche overlap	foraging behavior (plant use), visitation rates, abundance	-/0
Thomson 2016	*A*. mellifera*	*Bombus* spp.	North America (USA)	field (natural)	density	niche overlap	densities	-
Torne-Noguera et al. 2016	*A*. *mellifera*	many	Europe (Spain)	field (natural)	distance to apiary, visitation rate	resource consumption (nectar & pollen consumption)	visitation rate, wild bee biomass	-
Walther-Hellwig et al. 2006	*A*. *mellifera*	*Bombus* spp.	Europe (Germany)	field (agricultural)	density	tongue length	visitation rates/local abundance	-/0
Wilms & Weichers 1997	*A*. mellifera*	*Melipona bicolor*, *Melipona quadrifasciata*	South America (Brazil)	field (natural)	foraging behavior (types & amount of pollen & nectar collected)	niche overlap	foraging behavior (types & amount of pollen & nectar collected)	-

^1^ Commercial bumble bee colonies were used as indicators for conspecific wild bumble bees

* Indicates managed bee species that were used outside of their native range

**Table 2:** Studies published from 1900–2016 examining the potential effect of managed bees on wild bees through changes in plant communities, including the spread of exotic plants. For all studies, we recorded the species of managed and wild bees, and indicated whether managed bees were native or exotic to the study region, the location (continent and country) and context of the study including field (natural, semi-natural, developed, agricultural, or experimental plot), lab, or greenhouse, and all variables measured, including the managed bee metric (independent variable), plant metric (dependent variable), and any explanatory or mechanistic variables. The overall effect of managed bees on plant communities, as reported by the study, is also recorded and noted as positive (+), neutral (0), negative (-), or mixed.

Reference	Managed bee species (* indicates exotic range)	Wild bee species	Location	Context	Managed bee metric (independent variable)	Explanatory mechanism variable	Plant metric (dependent variable)	Reported effect
Abe et al. 2011	Apis mellifera*	*Xylocopa ogasawarensis* & others	Asia (Japan)	field (natural)	visitation rates	pollen limitation	fruit set	-
Aslan et al. 2016	*A*. mellifera*	many	North America (USA)	field (natural)	visitation rates	none	none	+/0
Barthell et al. 2001	*A*. mellifera*	many	North America (USA)	field (natural)	visitation rates	none	seed set	-
Beavon & Kelly 2012	*A*. mellifera*, *Bombus* spp.*	many	New Zealand	field (natural)	visitation rates, presence/absence	none	fruit set, seed set, fruit size, germination success	-
Bruckman & Campbell 2014	*A*. mellifera*	many	North America (USA)	field (natural)	visitation rates, foraging behavior (pollen deposition)	pollinator importance (visitation rates x conspecific pollen deposition)	seed set	+/0
Carbonari et al. 2009	*A*. mellifera*	none	South America (Brazil)	field (natural)	foraging behavior (frequency of nectar robbing)	occurrence of illegitimate visits	floral abortion	-
Cayuela et al. 2011	*A*. *mellifera*	none	Europe (Spain)	field (natural)	distance from apiary	none	fruit set	+/0
Chamberlain & Schlising 2008	*A*. mellifera*	many	North America (USA)	field (natural)	visitation rates	none	seed set	+
Descamps et al. 2015	*A*. *mellifera*	many	Europe (France)	field (natural)	visitation rates	none	none	+
Dick 2001	*A*. mellifera scutellata*	many	South America (Brazil)	field (natural)	visitation rates	none	seed set, genetic diversity, gene flow	+
Dohzono et al. 2008	Bombus terrestris*	*Bombus ardens*, *Bombus hypocrita*	Asia (Japan)	field (natural)	presence/absence	occurrence of illegitimate visits	fruit & seed set	-
Dupont et al. 2004	*A*. mellifera*	many	Canary Islands	field (natural)	abundance	foraging behavior (visitation length, foraging preferences)	seed set & viability	0
Esterio et al. 2013	*B*. terrestris*	many	South America (Chile)	field (natural)	visitation rates, foraging behavior (pollen collection, pollen deposition)	none	none	0
Faria & Araujo 2015	*A*. mellifera*	*Augochloropsis* spp.	South America (Brazil)	field (natural)	pollinator effectiveness (fruit set per visit)	none	fruit set	+
Faria & Araujo 2016	*A*. mellifera*	many	South America (Brazil)	field (natural)	visitation rates	none	none	+
Gilpin et al. 2014	*A*. mellifera*	many	Australia	field (natural)	visitation rates, foraging behavior (inter & intra-plant movements, pollen diversity on body)	pollinator fidelity	relative plant distribution	+
Goulson & Derwent 2004	*A*. mellifera*	many	Australia	field (natural), greenhouse	abundance, visitation rates, presence/absence	none	fruit set, seed set	-
Goulson & Rotheray 2012	*A*. mellifera*, *B*. terrestris*	many	Tasmania	field (natural)	visitation rates	none	population size, seed set	-/0
Gross 2001	*A*. mellifera*	many	Australia	field (natural)	abundance, visitation rates, foraging behavior (handling time)	none	pollen limitation, fruit set	+
Gross & Mackay 1998	*A*. mellifera*	many	Australia	field (natural)	visitation rates	pollen deposition & removal per visit	fruit & seed set	-
Gross et al. 2010	*A*. mellifera*	many	Australia	field (semi-natural, experiment plots)	visitation rates, abundance, presence/absence, foraging behavior (foraging time, number of probes per flower head, etc.)	none	abundance, seed set	-
Hanna et al. 2013	*A*. mellifera*	many	Hawaii	field (natural, experiment plots)	visitation rates, presence/absence	none	fruit set	+
Hingston 2005	*B*. terrestris*	none	Australia	field (garden)	visitation rates, foraging behavior (floral preferences)	none	none	0
Hermansen et al. 2014	*A*. mellifera*	many	Australia	field (natural)	visitation rates, foraging behavior (pollen load diversity, pollen removal & deposition)	none	none	+
Horskins & Turner 1999	*A*. mellifera*	many	Australia	field (natural)	foraging behavior (temporal foraging patterns, stigma contact, nectar vs. pollen collection, pollen load diversity)	none	none	+
Junker et al. 2010	*A*. mellifera*	*Hylaeus* spp.	Hawaii	field (natural)	presence/absence, visitation rates, foraging behavior (foraging trip duration, stigma contacts, resource collection)	pollinator effectiveness	fruit set	+
Kaiser-Bunbury & Müller 2009	*A*. mellifera*	many	Mauritius	field (experiment plots)	visitation rates	none	fruit set, seed set, fruit size & weight	+
Kaiser-Bunbury et al. 2011	*A*. mellifera*	many	Seychelles	field (natural)	visitation rates	none	plant reproductive success, fruit set	-/0
Kenta et al. 2007	Bombus terrestris*	*Bombus* spp.	Asia (Japan)	greenhouse	presence/absence	rate of legitimate floral visits	fruit set, fruit quality	-
Liu et al. 2013	*A*. mellifera*	many	Asia (China)	field (natural, experiment plots)	visitation rates	pollen transfer & deposition	fruit & seed set	-
Liu et al. 2006	*A*. mellifera*	many	North America (USA)	field (natural)	visitation rates	none	fruit set	-
Lomov et al. 2010	*A*. mellifera*	many	Australia	field (natural)	presence/absence, visitation rates, foraging behavior (contact with stigma & anthers)	pollen count per stigma, presence/absence of germinated pollen	fruit & seed set	0
Madjidian et al. 2008	Bombus ruderatus*	*Bombus dahlbomii*	South America (Argentina)	field (natural)	visitation rates, foraging behavior (time spent per flower, pollen deposition)	pollinator effectiveness (efficiency per visit*visitation frequency)	seed set	+
McGregor et al. 1959	*A*. mellifera*	many	North America (USA)	field (natural)	visitation rates, foraging behavior	none	none	+/0
Miller et al. 2015	*A*. mellifera*	*Hylaeus* spp.	Hawaii	field (natural/ semi-natural)	visitation rates, foraging behavior (pollen quantity, type & diversity on body)	none	none	-
Montalva et al. 2011	*B*. terrestris*, *B*. ruderatus*	*B*. *dahlbomii*, *Bombus funebris*	South America (Chile)	field (natural)	distribution, foraging behavior (floral association)	none	distribution	-
Morandin & Kremen 2013	*A*. mellifera*	many	North America (USA)	field (natural)	abundance, foraging behavior (floral preference)	none	none	+/0
Ott et al. 2016	*A*. mellifera*	*Bombus vosnesenskii*, *Xylocopa* spp.	North America (USA)	field (natural)	visitation rates, foraging behavior (handling time, contact with pollen/stigma, nectar intake), body size	none	none	0
Richardson et al. 2016	*A*. mellifera*	many	North America (USA)	field (natural)	visitation rates, foraging behavior (number of floral visits per plant, plant preferences)	none	numbers of seed capsules, intact seeds, & total seeds	+
Sanguinetti & Singer 2014	*A*. mellifera*, *B*.terrestris*, *B*. ruderatus*	many	South America (Argentina)	field (natural)	visitation rates, pollinator behavior (time per flower, number of flowers visited)	none	fruit set	+
Simpson et al. 2005	*A*. mellifera*	many	Australia	field (natural)	presence/absence, visitation rates, foraging behavior (flower tripping)	pollinator efficacy (fruit set per single visit)	seed set, fruit set	-
Stout et al. 2002	*A*. mellifera*, *B*. terrestris*	many	Tasmania	field (natural)	visitation rates	none	seed set, number of ovules fertilized per flower	-
Sun et al. 2013a	*A*. mellifera*, *B*. terrestris*	many	Asia (China)	field (natural)	visitation rates, foraging behavior (resource collection, number of flower visits per foraging bout, pollen removal & deposition)	pollination efficacy (combinations of all bee variables)	fruit & seed set	+
Sun et al. 2013b	*A*. mellifera*	many	Asia (China)	field (natural, experiment plots)	presence/absence, visitation rate, foraging behavior (number of capitula visited per plant, pollen load diversity)	none	seed set	+/0
Taylor & Whelan 1988	*A*. mellifera*	many	Australia	field (natural)	visitation rate, foraging behavior (nectar vs. pollen collection, pollen deposition, pollen type & diversity)	none	none	-
Woods et al. 2012	*A*. mellifera*	many	North America (USA)	field (natural)	visitation rate, foraging behavior	none	none	-
Xia et al. 2007	*A*. mellifera*, *Apis cerana*	*Bombus richardsi*, *Bombus*. *atrocinctus*	Asia (China)	field (natural)	presence/absence, abundance, visitation rate, foraging behaviors (intra- & inter- plant movement)	none	outcrossing rates, fruit & seed set	+

* Indicates managed bee species that were used outside of their native range

**Table 3:** Studies published from 1900–2016 examining the potential transmission of pathogens from managed to wild bees. For all studies, we recorded the species of managed and wild bees, and indicated whether managed bees were native or exotic to the study region, the location (continent and country) and context of the study including field (natural, semi-natural, developed, agricultural, or experimental plot), lab, or greenhouse, and all variables measured, including the managed bee metric (independent variable), wild bee metric (dependent variable), and any explanatory or mechanistic variables. The overall effect of managed bees on wild bees via pathogens, as reported by the study, is also recorded and noted as positive (+), neutral (0), negative (-), or mixed.

Reference	Managed bee species (* indicates non-native)	Wild bee species	Location	Context	Managed bee metric (independent variable)	Explanatory mechanism variable	Wild bee metric (response variable)	Reported effect
Arbetman et al. 2013	Bombus ruderatus*, Bombus terrestris*	*Bombus dahlbomii*	South America (Argentina)	field (natural)	presence/absence	none (transmission implied)	parasite presence/absence	-
Cameron et al. 2016	*Bombus* spp.	*Bombus* spp.	North America (USA)	lab	before/after pathogen introduction from commercial colonies	none (transmission implied)	pathogen prevalence, pathogen genetic variation	-
Colla et al. 2006	*Bombus impatiens*	*Bombus* spp.	North America (Canada)	field (semi-natural)	distance to commercial greenhouses	none (transmission implied)	pathogen prevalence	-
Dolezal et al. 2016	Apis mellifera*	many	North America (USA)	field (natural, agriculture), lab	pathogen prevalence, viral load	none	pathogen prevalence, pathogen load, lethality to bees	-/0
Forsgren et al. 2015	*A*. mellifera*	*Apis cerana*	Asia (Vietnam & China)	field	pathogen prevalence	none	pathogen prevalence	0
Fürst et al. 2014	*A*. *mellifera*	*Bombus* spp.	Europe (UK)	field, lab	pathogen prevalence	transmission	pathogen susceptibility/ infectivity, pathogen prevalence	-
Genersch et al. 2006	*A*. *mellifera*	*B*. *terrestris*, *Bombus pascuorum*	Europe (Germany)	field	presence	none (transmission implied)	pathogen occurrence	-
Gilliam et al. 1994	*A*. mellifera*^1^	*Xylocopa californica arizonensis*	North America (USA)	field (natural)	none	none (transmission implied)	pathogen occurrence	-
Graystock et al. 2013	*A*. *mellifera* ^1^	*Bombus* spp.	Europe (UK)	field, lab	none	none (transmission implied)	pathogen prevalence & infectivity	-
Graystock et al. 2014	*Bombus* spp., *A*. *mellifera*	*Bombus* spp.	Europe (UK)	field (agricultural)	presence/absence, distance from apiary	none	pathogen/parasite prevalence & richness	-
Hoffmann et al. 2008	*A*. mellifera*	*B*. *impatiens* ^2^	North America (USA)	greenhouse, lab	parasite host preference & host shifting	none	parasite host preference & host shifting, bee defense behavior	-
Koch & Strange 2012	*Bombus* spp. ^1^	*Bombus occidentalis*, *Bombus moderatus*	North America (USA)	field (natural)	none	none (transmission implied)	bee distribution & relative abundance, pathogen prevalence	0
Kojima et al. 2011	*A*. mellifera*	*A*. *cerana*	Asia (Japan)	field	infection frequency	none (implied transmission)	infection frequency	-/0
Levitt et al. 2013	*A*. mellifera*	many	North America (USA)	field (natural)	pathogen presence	none (implied transmission)	pathogen presence	-
Li et al. 2011	*A*. mellifera*^1^	*Bombus huntii*	North America (USA)	field, lab	none	none	pathogen infectivity	-
Maharramov et al. 2013	*B*. terrestris*, *B*. ruderatus*, *A*. mellifera*	*B*. *dahlbomii*	South America (Argentina)	field (natural)	genetic description of parasite	none (implied transmission)	genetic description of parasite	-
McMahon et al. 2015	*A*. *mellifera*	*Bombus* spp.	Europe (UK)	field	abundance (estimated), pathogen prevalence, pathogen load	none	pathogen prevalence, pathogen load	-
Murray et al. 2013	*B*. *terrestris*	*Bombus* spp.	Europe (Ireland)	field (agricultural)	pathogen prevalence	foraging behavior	pathogen prevalence	-
Niwa et al. 2004	*B*. terrestris*	*Bombus hypocrita*, *Bombus diversus*	Asia (Japan)	lab	pathogen prevalence	none	pathogen infectivity	-
Otterstater et al. 2008	*B*. *impatiens*	*Bombus* spp.	North America (Canada)	field (agricultural), lab	presence/absence, distance from greenhouse	none (implied transmission)	pathogen prevalence	-
Peng et al. 2011	*A*. mellifera*^1^	*B*. *huntii*	North America (USA)	field, lab	none	none	pathogen infectivity	-
Plischuk & Lange 2009	Bombus terrestris*	*Bombus atratus*, *Bombus morio*, *Bombus bellicosus*, *Bombus opifex*, *Bombus*. *tucumanus*	South America (Argentina)	field (natural)	pathogen prevalence	none (implied transmission risk)	pathogen prevalence	0
Plischuk et al. 2009	*A*. mellifera*^1^	*B*. *atratus*, *B*. *morio*, *B*. *bellicosus*	South America (Argentina)	field (natural)	none	none (implied transmission risk)	pathogen presence	-/0
Ravoet et al. 2014	*A*. *mellifera*	*Osmia* spp., *Andrena* spp., *Heriades truncorum*	Europe (Belgium)	field (developed)	pathogen presence	none	pathogen presence	-
Singh et al. 2010	*A*. mellifera*	many	North America (USA)	field (natural, agricultural), lab	pathogen presence	transmission	pathogen presence	-
Szabo et al. 2012	*B*. terrestris*	*Bombus affinis*, *Bombus terricola*, *Bombus pensylvanic-us*	North America	field (natural)	density of vegetable greenhouses	none (implied transmission)	bee geographic range (historic/current)	-/0
Whitehorn et al. 2013	*B*. *terrestris*, *B*. *terrestris audax*	*B*. *pascuorum*, *Bombus pratorum*, *Bombus lapidarius*	Europe (UK)	field (agricultural)	presence/absence	none (implied transmission)	pathogen prevalence & abundance	0

^1^ No measurement of managed bees taken; pathogen examined known to be specific to a managed bee species

^2^ Commercial bumble bee colonies were used as indicators for conspecific wild bumble bees

* Indicates managed bee species that were used outside of their native range

**Fig 3:**
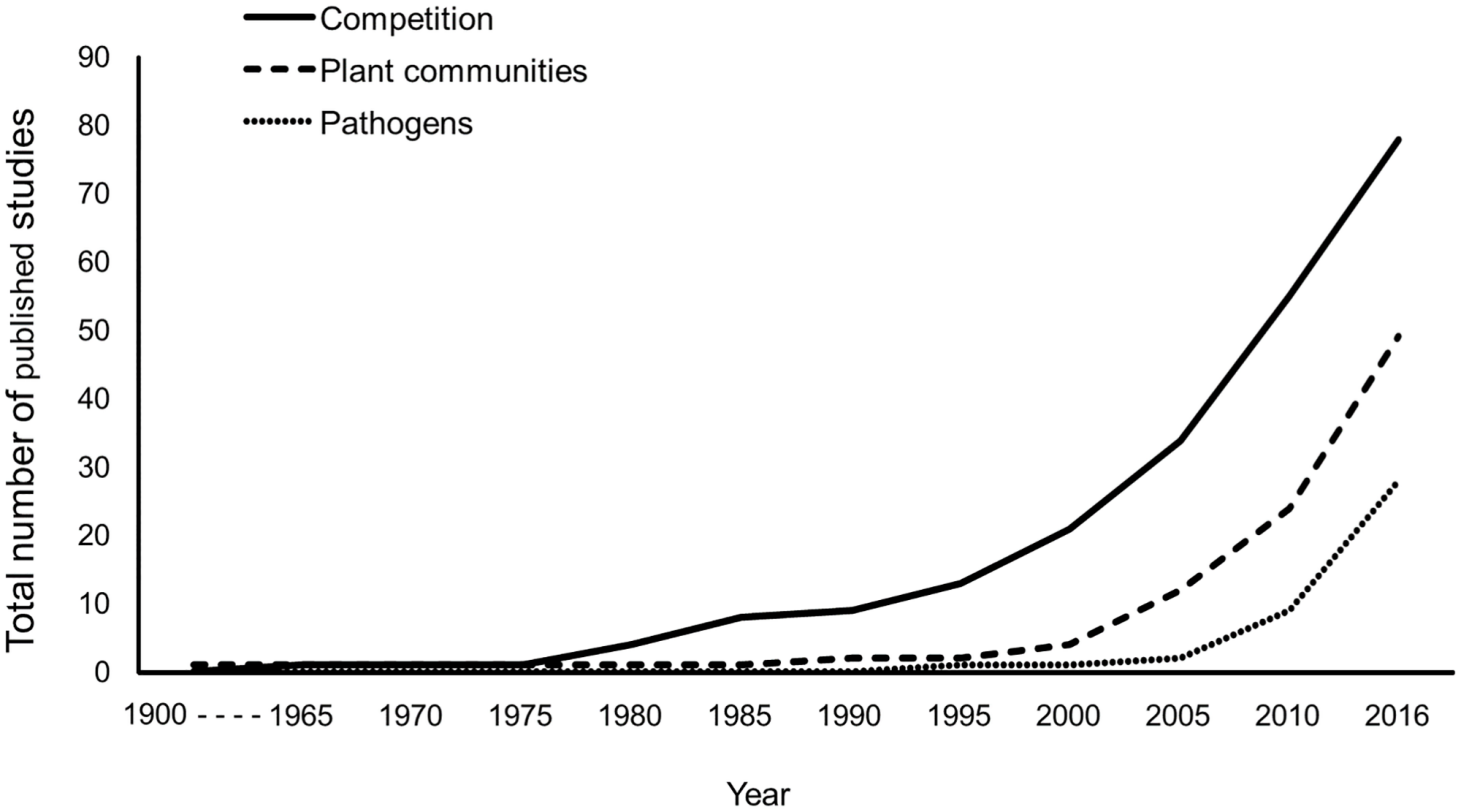
Publication trends. The total number of published studies over time from 1900–2016 that examined the effects of managed bees on wild bees via three reviewed mechanisms: competition for resources, changes in plant communities (specifically native and exotic plant populations), and transmission of pathogens. While the literature search began in 1900, the first publication within these topical areas did not occur until 1964.

Most competition studies were done in North America (n = 19) and Europe (n = 17), followed by South America (n = 14) and Asia (n = 12), with fewer done in Australia (n = 9), Africa (n = 4) or on smaller islands (n = 3) (Table 1). In contrast, of studies done on plant communities, the majority were conducted in Australia (n = 11) and North America (n = 10), followed by islands (n = 9), South America (n = 8), and Asia (n = 7), with few conducted in Europe (n = 2) and none in Africa (Table 2). Studies on pathogens were done primarily in North America (n = 12) and Europe (n = 8), with few in South America (n = 4) and Asia (n = 3), and none in Africa, Australia, or on smaller islands (Table 3). The vast majority of competition and plant studies were conducted in the field, specifically in natural/semi-natural habitats (69% and 85%, respectively, Tables 1 and 2). Pathogen studies were more variable, with many conducted in managed habitats including agricultural systems, or across multiple habitat types, or within the lab (Table 3). Studies on competition were published earlier and with greater frequency as compared to the other topical areas; competition studies began to be published at increasing rates around 1975, while studies on plant communities increased around 2000, and studies on pathogens were not published in notable numbers until 2005 (Fig 3).

### Competition

**Fig 4:**
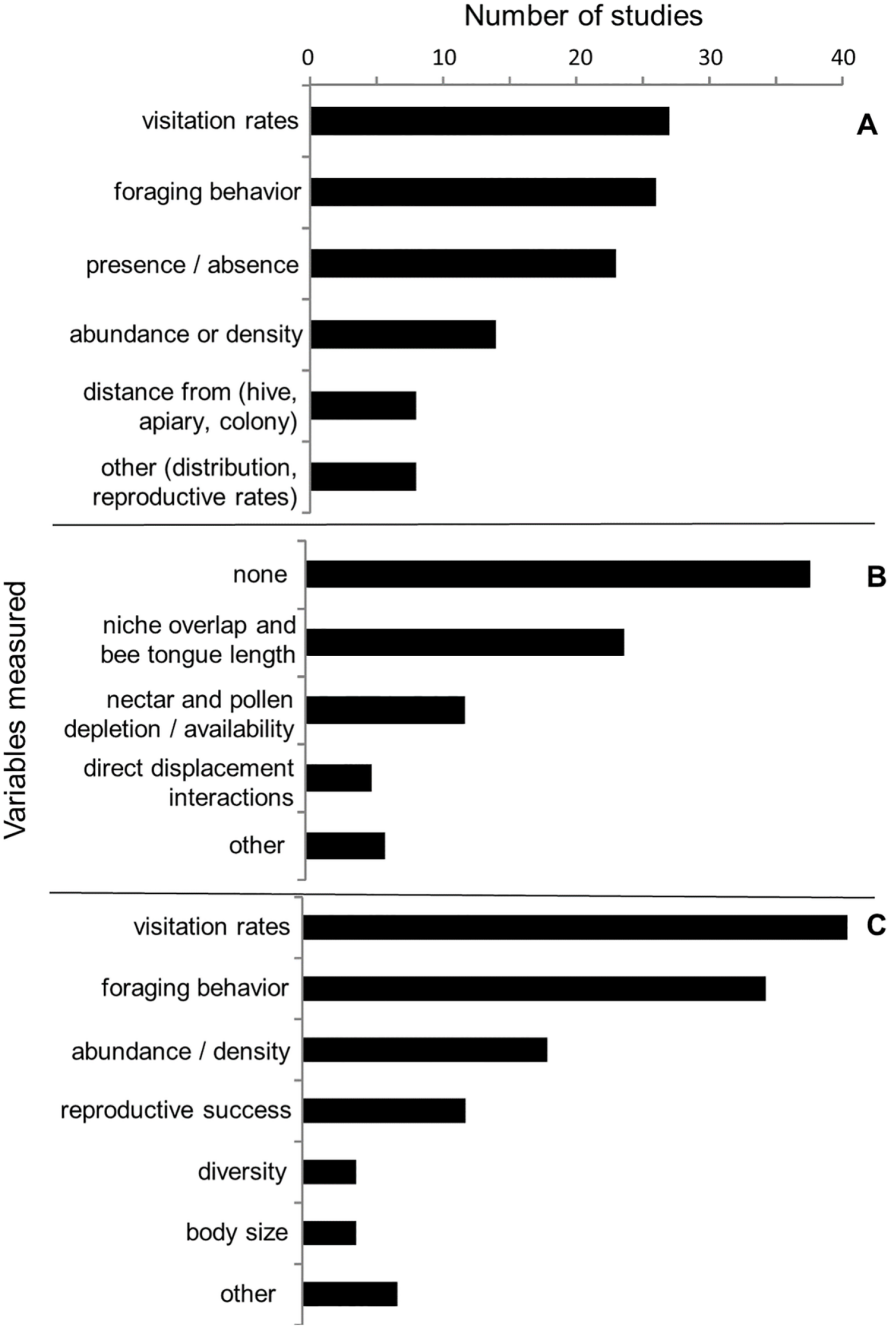
Reviewed effects of managed bees on wild bees through competition for shared resources. Variables reported by studies examining the competitive effects of managed bees on wild bees including (A) managed bee metrics (independent variables), (B) potential mechanisms (explanatory variables), and (C) wild bee responses (dependent variables).

Of the studies that examined competition between managed bees and wild bees, the most commonly measured independent variables associated with managed bees were visitation rates (n = 27) and various aspects of foraging behaviors such as handling time, pollen vs. nectar collection, or nectar robbing (n = 26), followed by presence/absence (n = 23), and abundance or density (n = 14). Fewer studies analyzed competition as a function of the distance from managed bee colonies (n = 8). The most commonly examined wild bee responses to managed bees were visitation rates to flowers (n = 40) and other aspects of bee foraging behaviors (n = 34), with fewer studies examining bee abundance or density (n = 18), bee reproductive success (n = 12), or bee diversity (n = 4) as a function of managed bees. The majority of studies (n = 38) did not measure explanatory variables, or potential mechanisms for the observed results, though some looked at the degree of niche overlap between managed and wild bees (n = 24), depletion or availability of nectar and pollen (n = 12), or direct displacement interactions between managed and wild bees (n = 5) (Fig 4A–4C).

**Fig 5:**
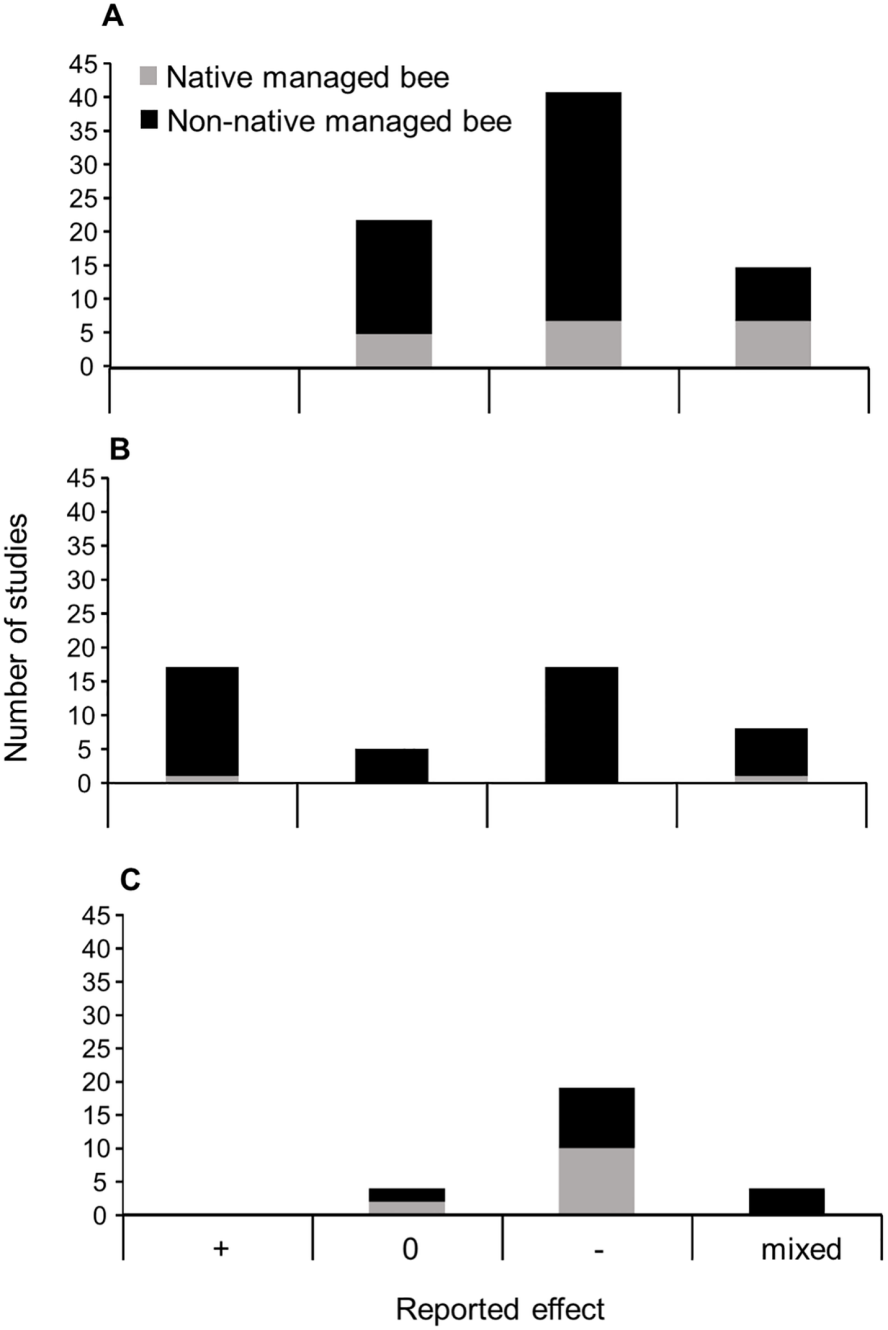
Reported results from reviewed studies on the effects of managed bees on wild bees. The total number of reviewed studies that found positive (+), neutral (0), negative (-), or mixed effects of managed bees on wild bees through (A) competition for shared resources, (B) changes in plant communities, and (C) transmission of pathogens. Studies within each category are divided into those that examined managed bees within their native range, and those that studied managed bees within their introduced range.

Fifty-three percent of studies reported a negative effect of managed bees on wild bees via competition for shared resources while 28% reported no effect and 19% reported mixed effects (Fig 5A). Though no studies reported entirely positive effects, some positive effects were included in studies reporting mixed effects (Table 1). Negative effects were more common with managed bees outside of their native range (58% of studies) as compared to managed bees within their native range (37%), indicating that the use of managed bees outside of their native range is more likely to have negative competitive effects on wild bees (Fig 5A).

### Plant communities

**Fig 6:**
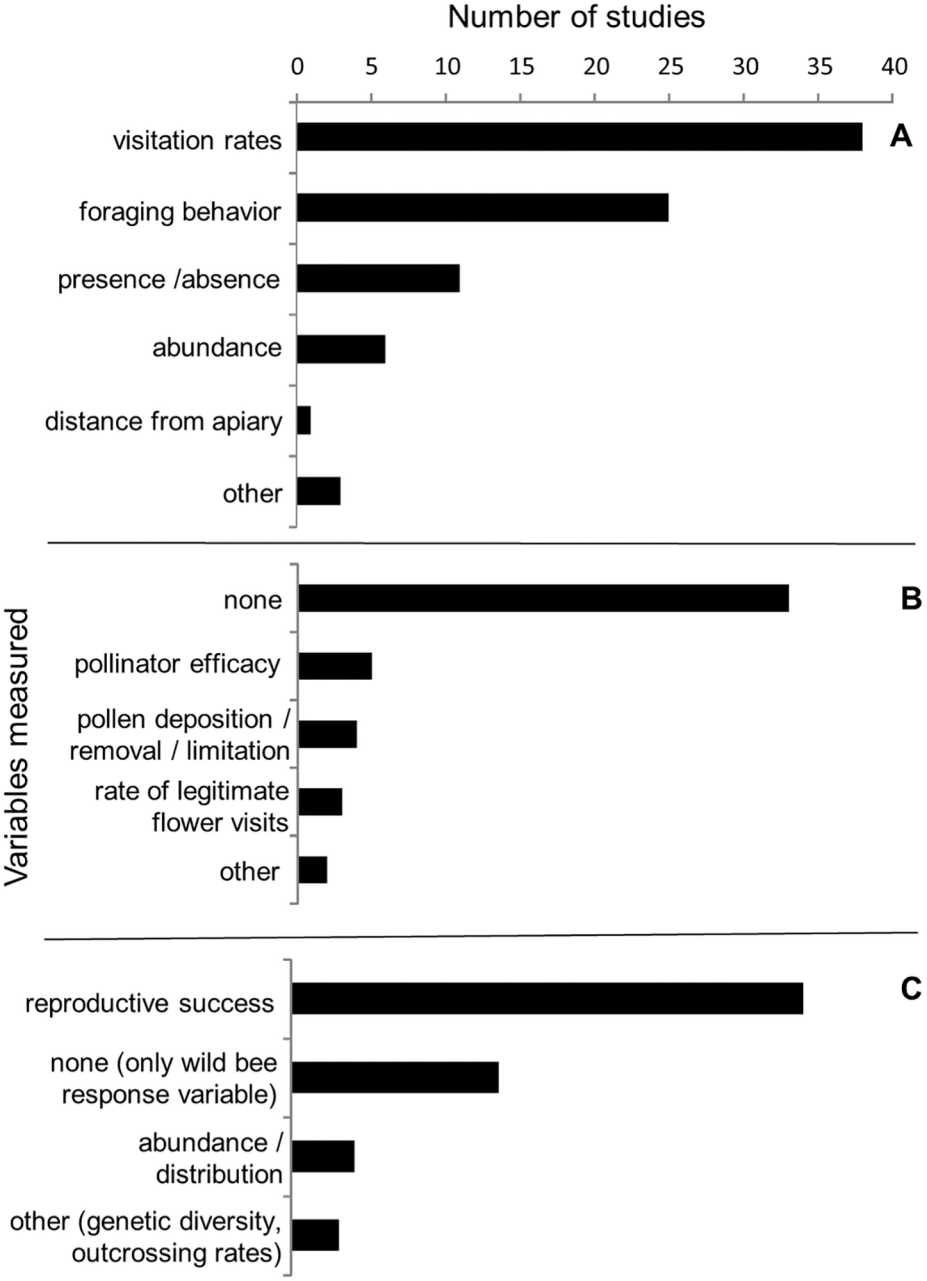
Reviewed effects of managed bees on wild bees through changes in plant communities. Variables reported by studies examining the effects of managed bees on plant communities including (A) managed bee metric (independent variable), (B) potential mechanism (explanatory variable), and (C) plant responses (dependent variable).

Among studies examining the potential effects of managed bees on wild bees through changes in plant communities, floral visitation rates were the most commonly measured independent variable associated with managed bees (n = 38) followed by other aspects of bee foraging behaviors (n = 25). Few studies examined plant responses as a function of managed bee presence/absence (n = 11), abundance (n = 6), or distance to managed bee colonies (n = 1). The majority of studies (n = 32) measured individual-level reproductive success of native or exotic plants as the response variable, such as fruit or seed set, while few studies (n = 4) examined population-level responses such as plant abundance or distribution. Most studies did not measure an explanatory or mechanistic variable, though a few studies measured pollen deposition or removal from managed bee visits (n = 4), or calculated pollinator efficacy (n = 5), a metric combining bee visitation rates, various aspects of bee foraging behavior, and/or plant reproductive success per pollinator visit (Fig 6A–6C).

An equal number of studies reported positive (36%) and negative (36%) effects of managed bees on native plant communities, with the remainder reporting mixed effects (17%) or no effects (11%) (Fig 5B). The vast majority of studies examined managed bees outside of their native range; only two studies examined managed bees within their native range, and these studies found positive or mixed effects of managed bees on native plant communities (Fig 5B).

### Pathogens

**Fig 7:**
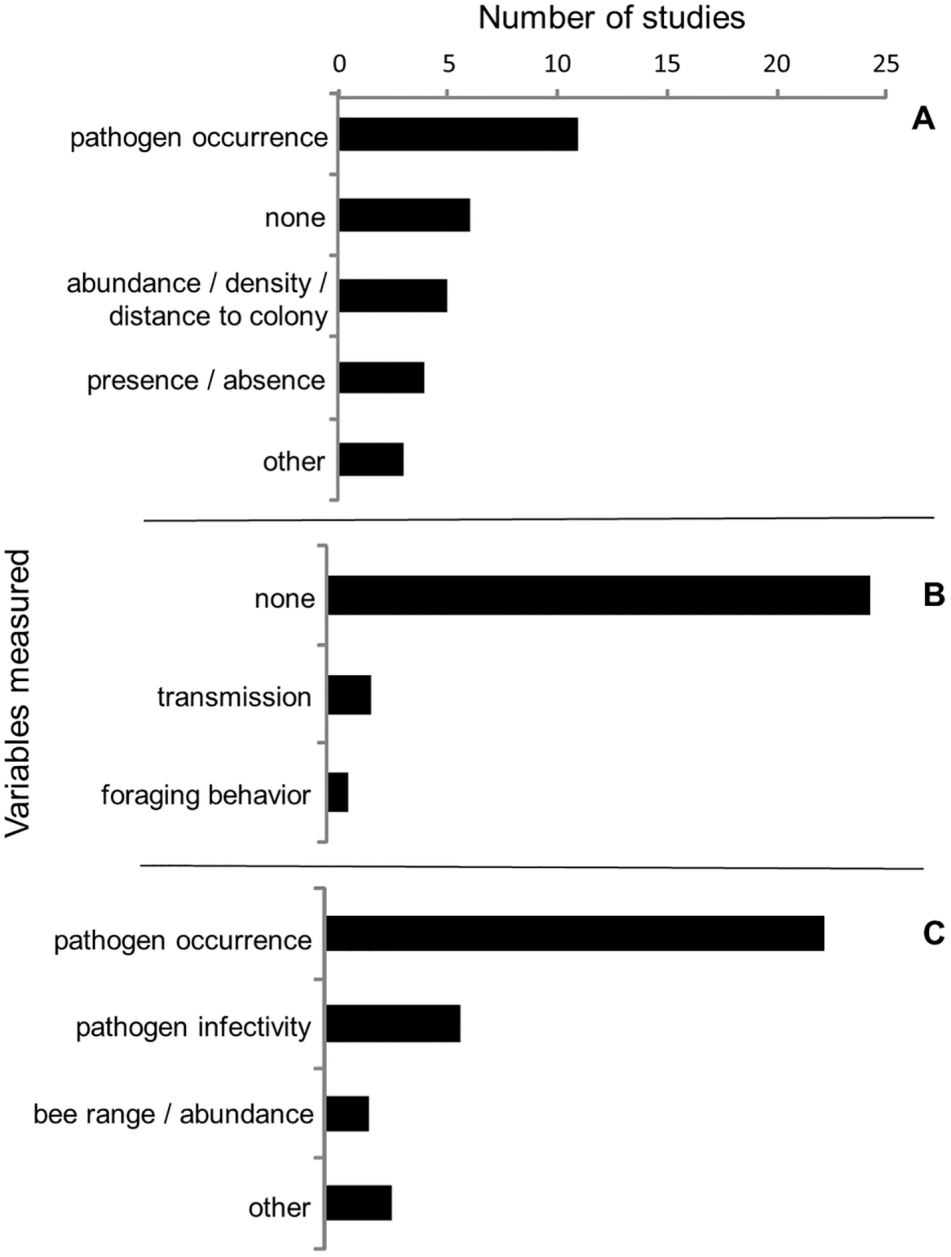
Reviewed effects of managed bees on wild bees through transmission of pathogens. Variables reported by studies examining the effects of managed bees on wild bees through pathogens including (A) managed bee metric (independent variable), (B) potential mechanisms (explanatory variable), and (C) wild bee responses (dependent variable).

Among studies examining the effect of managed bees on wild bees via transmission of pathogens, the occurrence of pathogens within managed bee populations was the most commonly measured independent variable, including the presence/absence of pathogens, frequency of pathogen detection within a population, and pathogen load or diversity per individual (n = 11). Fewer studies examined the effects of managed bees as a function of their abundance or density (n = 5) or presence/absence (n = 4). Furthermore, many studies did not measure any independent variable associated with managed bees (n = 6). That is, managed bees were assumed to occur in the study area or assumed to have a certain pathogen previously documented in other studies. The most commonly measured response variable was pathogen occurrence in wild bees (n = 22), followed by pathogen infectivity within wild bees (i.e. the ability of a pathogen to establish an infection) (n = 6), and with few studies measuring wild bee population-level responses such as wild bee abundance or geographic range (n = 2). The majority of studies (n = 24) did not measure potential mechanisms to explain their study findings and few (n = 2) documented transmission of pathogens from managed bees to wild bees (Fig 7A–7C).

The majority of studies (70%) reported negative effects of managed bees on wild bees via pathogen transmission, with 15% reporting no effects and an additional 15% reporting mixed effects. As compared to the other topical areas, studies on pathogen transmission more frequently examined managed bees within their native ranges. Of studies done with managed bees in their native ranges, a greater proportion found negative effects (83%) as compared to studies done with managed bees outside of their native ranges (60%), indicating that pathogen transmission and subsequent negative effects on wild bees may be as or more likely with managed bees used in their native ranges (Fig 5C).

## Discussion

In recent years, concern that managed bees have negative effects on wild bees has grown [3, 11, 12, 50], however no recent study has synthesized the research that examines these potential impacts. We found that across three mechanisms by which managed bees can affect wild bees (competition, changes in plant community composition, and pathogen transmission), the majority of studies concluded that managed bees have the potential to negatively affect wild bees. These conclusions may support the use of the precautionary principle when employing managed bees, particularly in or near areas with species of conservation concern. However, most of these studies did not measure wild bee fitness, population, or community-level responses including reproductive rates, survival, abundance, or diversity, making it difficult to draw long-term or broad-scale conclusions about the effects of managed bees. Furthermore, some studies found positive effects of managed bees, particularly on native plant communities, indicating that in some contexts, managed bees may aid in restoration or conservation efforts. These findings suggest that even after several decades of research on these topics, there remains some uncertainty as to the magnitude of the effects that managed bees have on wild bees.

Our review reaches some of the same conclusions as previous reviews on this topic, particularly with regards to competition, though our conclusions differ on other points due to both the expansion of the literature in recent years as well as our systematic approach to reviewing studies. Like the previous reviews [11, 12], we conclude that there is evidence for the presence of competition between managed bees and wild bees, though there is little evidence that this competition can lead to wild bee population declines. For instance, the majority of competition studies examined how managed bees affect wild bee foraging behaviors, in particular visitation rates to different flowers. How changes in wild bee foraging behaviors translate to variation in wild bee abundance or diversity was rarely studied. Since many bees are generalist flexible foragers and can partition resources in the presence of other bee species [23–24, 51], changes in foraging behaviors may not necessarily have population-level effects. In order to fully assess the effects of competition on wild bee populations, more studies that include measures of wild bee reproductive success or abundance as a function of managed bees are needed. While it may be more challenging to document long-term or direct effects of competition on wild bees, relatively recent studies provide good examples of how wild bee fitness or population-level responses can be evaluated [52–57].

Furthermore, the degree of competition and the subsequent effects on wild bee populations is likely to vary with the density of managed bees [58], which was not manipulated or observed in most studies (but see [52, 57, 59–62]). Studies that examined competition as a function of inferred managed bee density (e.g. variable distances from managed bee nests), found that competitive effects were strongest close to managed bee colonies, generally under 800 m, with reduced or no effects at increasing distances up to 1200 m suggesting that the impact of managed bees may be relatively local (< 1 km from the managed bee source) [52, 57, 60–61]. Additionally, the degree of competition may depend on overall resource availability, having significant effects on wild bees in contexts where resources are scarce, such as homogeneous landscapes, but insignificant effects during periods of high resource availability or in heterogeneous landscapes [57, 63, but see 76]. Therefore, while there is evidence that managed bees compete with wild bees for shared resources, in contexts with abundant resources, both managed and wild bee populations may be able to coexist.

While a previous review [11] concluded that the effects of managed bees on native plant communities were generally negative, we found an equal number of studies showing managed bees to be important pollinators of native plants as those that showed them to pollinate exotic invasive plants. However, as in the studies on competition, most plant community studies showed potential effects, both positive and negative, but did not show direct or long-term effects of managed bees on plant community composition. For example, some studies compared managed bee and wild bee foraging behaviors, in particular their preferences for native vs. exotic plants, but did not measure the effects of such preferences on plant reproduction, abundance, or diversity. Even among studies that measured plant reproductive output as a function of managed bees, individual-level responses such as fruit and seed set were not followed to population-level responses such as plant abundance or geographic range expansion (e.g., [29–30, 64–67], but see [68]). Furthermore, while it was generally outside the scope of these studies, the consequences of such changes in plant community composition for wild bees has not been well examined, and will likely vary across plant communities and bee species, especially between generalists and specialists [1, 69]. Thus, based on the literature we reviewed, the overall effects of managed bees on wild bees via changes in plant communities remains speculative.

Since the publication of previous reviews, research on pathogen transmission from managed bees to wild bees has increased rapidly, and with it, a greater focus on managed bumble bees in addition to managed honey bees. The conclusions reached by these studies primarily indicate negative effects of managed bees. However, these studies have similar limitations to those on the other topics, including that they do not show direct, long-term, or population and community-level effects of managed bees on wild bees. In particular, most studies documented the presence of shared pathogens in populations of managed and wild bees, but did not measure the effects of such pathogens on wild bees. Of the few studies that measured pathogen disease symptoms, infectivity, survival or fitness within wild bees, results varied across pathogens and were furthermore specific to controlled laboratory conditions [41, 70, 71]. Additional studies showed correlations between pathogen presence and wild bee species decline, however, in these cases, the origin of the pathogen is unclear and may not have come from managed bees [37, 72–73]. Furthermore, few studies documented transmission directionality making it unclear whether pathogens spilled over from managed bees to wild bees or the reverse. Thus, to demonstrate with more certainty the negative effects of pathogen transmission from managed bees to wild bees, future research should include experimental manipulative approaches to confirm transmission, and measure wild bee health, survival, or overall fitness with pathogens from managed bees. Nevertheless, the literature to date suggests that managed bees can transmit pathogens to wild bees [41], and that these pathogens may be contributing to wild bee population declines [50].

While our review found a substantial amount of research on the interactions between managed bees and wild bees, the relative effects of managed bees compared to factors such as habitat loss or pesticide exposure on wild bee populations are unknown and potentially confounding [12]. For example, it is difficult to examine the effects of managed bees in cropping systems independent of other aspects of agricultural management such as the use of pesticides or reduced plant diversity. Studies that control for these additional factors and compare wild bee responses in the presence/absence of managed bees, such as before-after-control-impact (BACI) analyses, or with varying densities of managed bees, are needed (e.g., [74–76]). Additionally, meta-analyses that compare the relative effects of different disturbances on wild bees would shed important insight on the role of managed bees in wild bee population declines. Currently, most meta-analyses have included factors related to habitat loss, habitat management, and fragmentation, but have not included the impact of managed bees [9–10, 77]. Understanding the relative magnitude of various disturbance factors is crucial for informing wild bee conservation priorities and the use of managed bees across both agricultural and natural habitats.

Finally, our review provides important insights on the relative risks of managed bees within and outside of their native ranges. While competition studies showed that managed bees outside of their native ranges are more likely to have negative effects on wild bees, studies on pathogen transmission suggest the opposite, with managed bees having greater negative effects on wild bees within their native ranges. Managed bees outside of their native ranges may have a competitive advantage over native wild bees due to reduced pressure from natural enemies [78–80]. Alternatively, managed bees within their native ranges may be more likely to transmit natural enemies to closely-related native wild species due to similarities in their foraging behaviors that could enhance transmission via flowers or direct contact [38, 40]. Additionally, wild populations may be more susceptible to pathogens or parasites transmitted by closely-related managed bees used within their native ranges in contrast to pathogens transmitted by distantly-related, exotic managed bees [45, 81–82]. Therefore, managed bees used both within and outside of their native ranges have the potential to affect wild bees, but the mechanisms responsible for such effects (i.e. competition versus pathogen transmission) may differ.

## Conclusions

Our review found that the majority of studies reach the conclusion that managed bees negatively affect, or have the potential to negatively affect, wild bees through competition, changes in plant communities, or transmission of pathogens. However, there was significant variability in study results, particularly in the areas of competition and plant communities, with some studies finding no or even positive effects of managed bees. We also found that many studies to date do not show direct or causal relationships between managed bees and wild bees. That is, studies lack controls or experimental manipulations, or do not measure critical parameters such as wild bee fitness, population-level, or community-level responses to managed bees. While such studies can be logistically challenging, thereby limiting their number, recent studies provide examples of novel approaches, large-scale experiments, and/or the use of long-term data in order to better understand the effects of managed bees [41, 54, 58, 63, 74–76, 82–87]. The conclusions of these recent, more comprehensive studies largely mirror the conclusions of the literature as a whole: competition studies were highly variable (55% reporting negative effects, 33% no effects, and 11% mixed effects), studies on pathogens provide strong evidence for the transmission of pathogens between managed and wild bees, but the effects of these pathogens on wild bee health and fitness are variable and/or unknown, and the effects of managed bees on native plant populations can be positive in some contexts.

Managed bees provide benefits to humans, including crop pollination, and these benefits may outweigh the risks to native ecosystems in some cases. In order to limit the impact of managed bees, public land managers should consider site-specific attributes such as the species of managed bee and whether it is native to the region, the proposed densities of managed bees, relative resource availability (i.e. landscape diversity), whether managed bee colonies have been evaluated for pathogens and parasites, and whether there are declining wild bee species of conservation concern in the region before allowing managed bees on public lands. Commercial bee producers, including rearing centers, can furthermore limit the impact of managed bees by frequent screening for and treatment of pathogens. Industry guidelines that regulate the movement of managed bees across large regions will reduce the potential for pathogen introduction and spread. Finally, growers that use managed bees in greenhouse contexts could limit negative effects by ensuring that managed bees cannot escape to the wild, and growers that use managed bees in field settings may be able to reduce their impact by placing colonies in the center of agricultural fields or at maximum distances from natural habitats.

## Supporting information

S1 Checklist PRISMA checklist for systematic reviews. (DOCX)

S1 References Reference list of all studies included in this systematic review. (DOCX)
