# Supplementary material for: Do managed bees have negative effects on wild bees?: A systematic review of the literature
Source: PLoS One. 2017 Dec 8;12(12):e0189268. doi: 10.1371/journal.pone.0189268 (PMC5722319; doi:10.1371/journal.pone.0189268)
Supplement: S1 References — (DOCX) [file pone.0189268.s002.docx]

**S1 References. Reference list of all studies included in this systematic review**

Abe, T., K. Wada, Y. Kato, S. Makino, and I. Okochi. 2011. Alien pollinator promotes invasive mutualism in an insular pollination system. Biological Invasions 13: 957–967.

Aizen, M. A., M. Lozada, and C. L. Morales. 2011. Comparative nectar-foraging behaviors and efficiencies of an alien and a native bumble bee. Biological Invasions 13: 2901–2909.

Aizen, M., and P. Feinsinger. 1994. Habitat fragmentation, native insect pollinators, and feral honey-bees. Ecological Applications 4: 378–392.

Arbetman, M. P., I. Meeus, C. L. Morales, M. A. Aizen, and G. Smagghe. 2013. Alien parasite hitchhikes to Patagonia on invasive bumblebee. Biological Invasions 15: 489–494.

Aslan, C. E., C. T. Liang, B. Galindo, K. Hill, and W. Topete. 2016. The role of honey bees as pollinators in natural areas. Natural Areas Journal 36: 478–488.

Badano, E. I., and C. H. Vergara. 2011. Potential negative effects of exotic honey bees on the diversity of native pollinators and yield of highland coffee plantations. Agricultural and Forest Entomology 13: 365–372.

Balfour, N. J., S. Gandy, and F. L. W. Ratnieks. 2015. Exploitative competition alters bee foraging and flower choice. Behavioral Ecology and Sociobiology 69: 1731–1738.

Balfour, N. J., M. Garbuzov, and F. L. W. Ratnieks. 2013. Longer tongues and swifter handling: why do more bumble bees (*Bombus spp.*) than honey bees (*Apis mellifera*) forage on lavender (*Lavandula spp.*)? Ecological Entomology 38: 323–329.

Barthell, J. F., J. M. Randall, R. W. Thorp, and A. M. Wenner. 2001. Promotion of seed set in yellow star-thistle by honey bees: evidence of an invasive mutualism. Ecological Applications 11: 1870–1883.

Bartomeus, I., M. Vila, and I. Steffan-Dewenter. 2010. Combined effects of *Impatiens glandulifera* invasion and landscape structure on native plant pollination. Journal of Ecology 98: 440–450.

Batra, S. W. T. 1999. Native bees (Hymenoptera: Apoidea) in native trees: *Nyssa sylvatica* Marsh. (Cornaceae). Proceedings of the Entomological Society of Washington 101: 449–457.

Beavon, M. A., and D. Kelly. 2012. Invasional meltdown: pollination of the invasive *Liana* *passiflora tripartita var. mollissima* (Passifloraceae) in New Zealand. New Zealand Journal of Ecology 36: 100–107.

Bruckman, D., and D. R. Campbell. 2014. Floral neighborhood influences pollinator assemblages and effective pollination in a native plant. Oecologia 176: 465–476.

Cameron, S. A., H. C. Lim, J. D. Lozier, M. A. Duennes, and R. Thorp. 2016. Test of the invasive pathogen hypothesis of bumble bee decline in North America. Proceedings of the National Academy of Sciences USA 113: 4386–4391.

Cane, J. H., and V. J. Tepedino. 2017. Gauging the effect of honey bee pollen collection on native bee communities. Conservation Letters 10: 205-210.

Carbonari, V., L. P. Polatto, and V. V. Aives-Junior. 2009. Evaluation of the impact on *Pyrostegia venusta* (Bignoniaceae) flowers due to nectar robbery by *Apis mellifera* (Hymenoptera, Apidae). Sociobiology 54: 373–382.

Carneiro, L. T., and C. F. Martins. 2012. Africanized honey bees pollinate and preempt the pollen of *Spondias mombin* (Anacardiaceae) flowers. Apidologie 43: 474–486.

Cayuela, L., S. Ruiz-Arriaga, and C. P. Ozers. 2011. Honeybees increase fruit set in native plant species important for wildlife conservation. Environmental Management 48: 910–919.

Chamberlain, S. A., and R. A. Schlising. 2008. Role of honey bees (Hymenoptera: Apidae) in the pollination biology of a California native plant, *Triteleia laxa* (Asparagales: Themidaceae). Environmental Entomology 37: 808–816.

Colla, S. R., M. C. Otterstatter, R. J. Gegear, and J. D. Thomson. 2006. Plight of the bumble bee: pathogen spillover from commercial to wild populations. Biological Conservation 129: 461–467.

Conner, J., and R. Neumeier. 1995. Effects of black mustard population-size on the taxonomic composition of pollinators. Oecologia 104: 218–224.

Descamps, C., L. Moquet, M. Migon, and A.-L. Jacquemart. 2015. Diversity of the insect visitors on *Calluna vulgaris* (Ericaceae) in Southern France heathlands. Journal of Insect Science 15: e130.

Dick, C. W. 2001. Genetic rescue of remnant tropical trees by an alien pollinator. Proceedings of the Royal Society B-Biological Sciences 268: 2391–2396.

Dohzono, I., Y. K. Kunitake, J. Yokoyama, and K. Goka. 2008. Alien bumble bee affects native plant reproduction through interactions with native bumble bees. Ecology 89: 3082–3092.

Dolezal, A. G., S. D. Hendrix, N. A. Scavo, J. Carrillo-Tripp, M. A. Harris, M. J. Wheelock, M. E. O’Neal, and A. L. Toth. 2016. Honey bee viruses in wild bees: viral prevalence, loads, and experimental inoculation. PLoS One 11 (11): e0166190.

Dupont, Y. L., D. M. Hansen, A. Valido, and J. M. Olesen. 2004. Impact of introduced honey bees on native pollination interactions of the endemic *Echium wildpretii* (Boraginaceae) on Tenerife, Canary Islands. Biological Conservation 118: 301–311.

El Shafie, H. A. F., J. B. B. Mogga, and T. Basedow. 2002. Studies on the possible competition for pollen between the honey bee, *Apis mellifera sudanensis*, and the imported dwarf honey bee *Apis florea* (Hym., Apidae) in North-Khartoum (Sudan). Journal of Applied Entomology-Zeitschrift Fur Angewandte Entomologie 126: 557–562.

Elbgami, T., W. E. Kunin, W. O. H. Hughes, and J. C. Biesmeijer. 2014. The effect of proximity to a honeybee apiary on bumblebee colony fitness, development, and performance. Apidologie 45: 504–513.

Esterio, G., R. Cares-Suarez, C. Gonzalez-Browne, P. Salinas, G. Carvallo, and R. Medel. 2013. Assessing the impact of the invasive buff-tailed bumblebee (*Bombus terrestris*) on the pollination of the native Chilean herb *Mimulus luteus*. Arthropod-Plant Interactions 7: 467–474.

Faria, R. R., and A. C. Araujo. 2015. Fruit set of distylous *Psychotria carthagenensis* Jacq. (Rubiaceae) mediated by *Apis mellifera* (Apidae) and species of *Augochloropsis* (Halictidae). Acta Botanica Brasilica 29: 278–281.

Faria, R. R., and A. C. Araujo. 2016. Flowering phenology and floral visitors in distylous populations of *Psychotria carthagenensis* (Rubiaceae) in Brazilian Cerrado. Annals of the Missouri Botanical Garden 101: 636–647.

Forup, M. L., and J. Memmott. 2005. The relationship between the abundances of bumblebees and honeybees in a native habitat. Ecological Entomology 30: 47–57.

Forsgren, E., W. Shi, G. L. Ding, Z. G. Liu, T. V. Tran, P. T. Tang, T. A. Truong, T. Q. Dinh, and I. Fries. 2015. Preliminary observations on possible pathogen spill-over from *Apis mellifera* to *Apis cerana*. Apidologie 46: 265–275.

Franco, E. L., C. M. L. Aguiar, V. S. Ferreira, and P. L. de Oliveira-Reboucas. 2009. Plant use and niche overlap between the introduced honey bee (*Apis mellifera*) and the native bumblebee (*Bombus atratus*) (Hymenoptera: Apidae) in an area of tropical mountain vegetation in northeastern Brazil. Sociobiology 53: 141–150.

Fürst, M. A., D. P. McMahon, J. L. Osborne, R. J. Paxton, and M. J. F. Brown. 2014. Disease associations between honeybees and bumblebees as a threat to wild pollinators. Nature 506: 364–366.

Genersch, E., C. Yue, I. Fries, and J. R. de Miranda. 2006. Detection of Deformed Wing Virus, a honey bee viral pathogen, in bumble bees (*Bombus terrestris* and *Bombus pascuorum*) with wing deformities. Journal of Invertebrate Pathology 91: 61–63.

Gilliam, M., B. J. Lorenz, and S. L. Buchmann. 1994. *Ascosphaera apis*, the chalkbrood pathogen of the honey bee, *Apis mellifera*, from larvae of a carpenter bee, *Xylocopa californica arizonensis*. Journal of Invertebrate Pathology 63: 307–309.

Gilpin, A.-M., D. J. Ayre, and A. J. Denham. 2014. Can the pollination biology and floral ontogeny of the threatened *Acacia carneorum* explain its lack of reproductive success? Ecological Research 29: 225–235.

Ginsberg, H. S. 1983. Foraging ecology of bees in an old field. Ecology 64: 165–175.

Goras, G., C. Tananaki, M. Dimou, T. Tscheulin, T. Petanidou, and A. Thrasyvoulou. 2016. Impact of honey bee (*Apis mellifera* L.) density on wild bee foraging behaviour. Journal of Apicultural Science 60: 49–61.

Goulson, D., and L. C. Derwent. 2004. Synergistic interactions between an exotic honeybee and an exotic weed: pollination of *Lantana camara* in Australia. Weed Research 44: 195–202.

Goulson, D., W. O. H. Hughes, L. C. Derwent, and J. C. Stout. 2002. Colony growth of the bumblebee, *Bombus terrestris*, in improved and conventional agricultural and suburban habitats. Oecologia 130: 267–273.

Goulson, D., and E. L. Rotheray. 2012. Population dynamics of the invasive weed *Lupinus arboreus* in Tasmania, and interactions with two non-native pollinators. Weed Research 52: 535–541.

Goulson, D., and K. R. Sparrow. 2009. Evidence for competition between honeybees and bumblebees; effects on bumblebee worker size. Journal of Insect Conservation 13: 177–181.

Graystock, P., D. Goulson, and W. O. H. Hughes. 2014. The relationship between managed bees and the prevalence of parasites in bumblebees. PeerJ 2: e522.

Graystock, P., K. Yates, B. Darvill, D. Goulson, and W. O. H. Hughes. 2013a. Emerging dangers: deadly effects of an emergent parasite in a new pollinator host. Journal of Invertebrate Pathology 114: 114–119.

Graystock, P., K. Yates, S. E. F. Evison, B. Darvill, D. Goulson, and W. O. H. Hughes. 2013b. The Trojan hives: pollinator pathogens, imported and distributed in bumblebee colonies. Journal of Applied Ecology 50: 1207–1215.

Gross, C. L. 2001. The effect of introduced honeybees on native bee visitation and fruit-set in *Dillwynia juniperina* (Fabaceae) in a fragmented ecosystem. Biological Conservation 102: 89–95.

Gross, C. L., L. Gorrell, M. J. Macdonald, and M. Fatemi. 2010. Honeybees facilitate the invasion of *Phyla canescens* (Verbenaceae) in Australia - no bees, no seed! Weed Research 50: 364–372.

Gross, C. L., and D. Mackay. 1998. Honeybees reduce fitness in the pioneer shrub *Melastoma affine* (Melastomataceae). Biological Conservation 86: 169–178.

Hanna, C., D. Foote, and C. Kremen. 2013. Invasive species management restores a plant–pollinator mutualism in Hawaii. Journal of Applied Ecology 50: 147–155.

Herbertsson, L., S. A. M. Lindstrom, M. Rundlöf, R. Bornmarco, and H. G. Smith. 2016. Competition between managed honeybees and wild bumblebees depends on landscape context. Basic and Applied Ecology 17: 609–616.

Hermansen, T. D., D. R. Britton, D. J. Ayre, and T. E. Minchinton. 2014. Identifying the real pollinators? Exotic honeybees are the dominant flower visitors and only effective pollinators of *Avicennia marina* in Australian temperate mangroves. Estuaries and Coasts 37: 621–635.

Hingston, A. B. 2005. Does the introduced bumblebee, *Bombus terrestris* (Apidae), prefer flowers of introduced or native plants in Australia? Australian Journal of Zoology 53: 29–34.

Hingston, A. B., and P. B. McQuillan. 1998. Does the recently introduced bumblebee *Bombus terrestris* (Apidae) threaten Australian ecosystems? Australian Journal of Ecology 23: 539–549.

Hingston, A. B., and P. B. McQuillan. 1999. Displacement of Tasmanian native megachilid bees by the recently introduced bumblebee *Bombus terrestris* (Linnaeus, 1758) (Hymenoptera: Apidae). Australian Journal of Zoology 47: 59–65.

Hoffmann, D., J. S. Pettis, and P. Neumann. 2008. Potential host shift of the small hive beetle (*Aethina tumida*) to bumblebee colonies (*Bombus impatiens*). Insectes Sociaux 55: 153–162.

Holmes, F.O. 1964. The distribution of honey bees and bumblebees on nectar-secreting plants. American Bee Journal January:12-13.

Horskins, K., and V. B. Turner. 1999. Resource use and foraging patterns of honeybees, *Apis mellifera*, and native insects on flowers of *Eucalyptus costata*. Australian Journal of Ecology 24: 221–227.

Hudewenz, A., and A.-M. Klein. 2013. Competition between honey bees and wild bees and the role of nesting resources in a nature reserve. Journal of Insect Conservation 17: 1275–1283.

Hudewenz, A., and A.-M. Klein. 2015. Red mason bees cannot compete with honey bees for floral resources in a cage experiment. Ecology and Evolution 5: 5049–5056.

Inari, N., T. Nagamitsu, T. Kenta, K. Goka, and T. Hiura. 2005. Spatial and temporal pattern of introduced *Bombus terrestris* abundance in Hokkaido, Japan, and its potential impact on native bumblebees. Population Ecology 47: 77–82.

Ings, T. C., N. L. Ward, and L. Chittka. 2006. Can commercially imported bumble bees out-compete their native conspecifics? Journal of Applied Ecology 43: 940–948.

Inoue, M. N., T. T. Makino, J. Yokoyama, and S. Sakai. 2010. Is *Bombus terrestris* (Hymenoptera: Apidae) a stronger competitor against native Japanese species? A comparison of foraging efficiency. Applied Entomology and Zoology 45: 71–75.

Inoue, M. N., and J. Yokoyama. 2010. Competition for flower resources and nest sites between *Bombus terrestris* (L.) and Japanese native bumblebees. Applied Entomology and Zoology 45: 29–35.

Ishii, H. S., T. Kadoya, R. Kikuchi, S.-I. Suda, and I. Washitani. 2008. Habitat and flower resource partitioning by an exotic and three native bumble bees in central Hokkaido, Japan. Biological Conservation 141: 2597–2607.

Junker, R. R., R. Bleil, C. C. Daehler, and N. Bluethgen. 2010. Intra-floral resource partitioning between endemic and invasive flower visitors: consequences for pollinator effectiveness. Ecological Entomology 35: 760–767.

Kaiser-Bunbury, C. N., and C. B. Müller. 2009. Indirect interactions between invasive and native plants via pollinators. Naturwissenschaften 96: 339–346.

Kaiser-Bunbury, C. N., T. Valentin, J. Mougal, D. Matatiken, and J. Ghazoul. 2011. The tolerance of island plant-pollinator networks to alien plants. Journal of Ecology 99: 202–213.

Kajobe, R. 2007. Pollen foraging by *Apis mellifera* and stingless bees *Meliponula bocandei* and *Meliponula nebulata* in Bwindi Impenetrable National Park, Uganda. African Journal of Ecology 45: 265–274.

Kato, M., and A. Kawakita. 2004. Plant-pollinator interactions in New Caledonia influenced by introduced honey bees. American Journal of Botany 91: 1814–1827.

Kato, M., A. Shibata, T. Yasui, and H. Nagamasu. 1999. Impact of introduced honeybees, *Apis mellifera*, upon native bee communities in the Bonin (Ogasawara) Islands. Researches on Population Ecology 41: 217–228.

Kenta, T., N. Inari, T. Nagamitsu, K. Goka, and T. Hiura. 2007. Commercialized European bumblebee can cause pollination disturbance: an experiment on seven native plant species in Japan. Biological Conservation 134: 298–309.

Koch, J. B., and J. P. Strange. 2012. The status of *Bombus occidentalis* and *B. moderatus* in Alaska with special focus on *Nosema bombi* incidence. Northwest Science 86: 212–220.

Kojima, Y., T. Toki, T. Morimoto, M. Yoshiyama, K. Kimura, and T. Kadowaki. 2011. Infestation of Japanese native honey bees by tracheal mite and virus from non-native European honey bees in Japan. Microbial Ecology 62: 895–906.

Kuhn, J., A. Hamm, M. Schindler, and D. Wittmann. 2006. Resource partitioning between the oligolectic leafcutter bee *Megachile lapponica* (Hymenoptera, Apiformes) and other visitors on flowers of *Epilobium angustifolium* (Onagracea). Mitteilungen der Deutschen Gesellschaft fur allgemeine und angewandte Entomologie 15: 389-392.

Levitt, A. L., R. Singh, D. L. Cox-Foster, E. Rajotte, K. Hoover, N. Ostiguy, and E. C. Holmes. 2013. Cross-species transmission of honey bee viruses in associated arthropods. Virus Research 176: 232–240.

Li, J., W. Peng, J. Wu, J. P. Strange, H. Boncristiani, and Y. Chen. 2011. Cross-species infection of deformed wing virus poses a new threat to pollinator conservation. Journal of Economic Entomology 104: 732–739.

Lindstrom, S. A. M., L. Herbertsson, M. Rundlöf, R. Bommarco, and H. G. Smith. 2016. Experimental evidence that honeybees depress wild insect densities in a flowering crop. Proceedings of the Royal Society B-Biological Sciences 283: 20161641

Liu, H., R. W. Pemberton, and P. Stiling. 2006. Native and introduced pollinators promote a self-incompatible invasive woody vine (*Paederia foetida* L) in Florida. Journal of the Torrey Botanical Society 133: 304–311.

Liu, W., Y. Wang, Q. Chen, and S. Yu. 2013. Pollination of invasive *Eichhornia crassipes* (Pontederiaceae) by the introduced honeybee (*Apis mellifera* L.) in South China. Plant Systematics and Evolution 299: 817–825.

Lomov, B., D. A. Keith, and D. F. Hochuli. 2010. Pollination and plant reproductive success in restored urban landscapes dominated by a pervasive exotic pollinator. Landscape and Urban Planning 96: 232–239.

Lye, G. C., S. N. Jennings, J. L. Osborne, and D. Goulson. 2011. Impacts of the use of nonnative commercial bumble bees for pollinator supplementation in raspberry. Journal of Economic Entomology 104: 107–114.

Madjidian, J. A., C. L. Morales, and H. G. Smith. 2008. Displacement of a native by an alien bumblebee: lower pollinator efficiency overcome by overwhelmingly higher visitation frequency. Oecologia 156: 835–845.

Maharramov, J., I. Meeus, K. Maebe, M. Arbetman, C. Morales, P. Graystock, W. O. H. Hughes, S. Plischuk, C. E. Lange, D. C. de Graaf, N. Zapata, J. J. Perez de la Rosa, T. E. Murray, M. J. F. Brown, and G. Smagghe. 2013. Genetic variability of the neogregarine *Apicystis bombi*, an etiological agent of an emergent bumblebee disease. PLoS ONE 8: e81475.

Martins, D. J. 2004. Foraging patterns of managed honeybees and wild bee species in an arid African environment: ecology, biodiversity and competition. International Journal of Tropical Insect Science 24: 105–115.

McGregor, S. E., S. M. Alcorn, E. B. Kurtz, and G. D. Butler. 1959. Bee visitors to saguaro flowers. Journal of Economic Entomology 52: 1002–1004.

McMahon, D. P., M. A. Furst, J. Caspar, P. Theodorou, M. J. F. Brown, and R. J. Paxton. 2015. A sting in the spit: widespread cross-infection of multiple RNA viruses across wild and managed bees. Journal of Animal Ecology 84: 615–624.

Menezes, C., C. I. da Silva, R. B. Singer, and W. E. Kerr. 2007. Competition among bees during foraging on *Schefflera arboricola* (Hayata) Merr. Bioscience Journal 23: 63 - 69.

Miller, A. E., B. J. Brosi, K. Magnacca, G. C. Daily, and L. Pejchar. 2015. Pollen carried by native and nonnative bees in the large-scale reforestation of pastureland in Hawai’i: implications for pollination. Pacific Science 69: 67–79.

Montalva, J., L. Dudley, M. Kalin Arroyo, H. Retamales, and A. H. Abrahamovich. 2011. Geographic distribution and associated flora of native and introduced bumble bees (*Bombus spp.*) in Chile. Journal of Apicultural Research 50: 11–21.

Morales, C. L., M. P. Arbetman, S. A. Cameron, and M. A. Aizen. 2013. Rapid ecological replacement of a native bumble bee by invasive species. Frontiers in Ecology and the Environment 11: 529–534.

Morandin, L. A., and C. Kremen. 2013. Bee preference for native versus exotic plants in restored agricultural hedgerows. Restoration Ecology 21: 26–32.

Murray, T. E., M. F. Coffey, E. Kehoe, and F. G. Horgan. 2013. Pathogen prevalence in commercially reared bumble bees and evidence of spillover in conspecific populations. Biological Conservation 159: 269–276.

Nagamitsu, T., T. Kenta, N. Inari, H. Horita, K. Goka, and T. Hiura. 2007a. Foraging interactions between native and exotic bumblebees: enclosure experiments using native flowering plants. Journal of Insect Conservation 11: 123–130.

Nagamitsu, T., T. Kenta, N. Inari, E. Kato, and T. Hiura. 2007b. Abundance, body size, and morphology of bumblebees in an area where an exotic species, *Bombus terrestris*, has colonized in Japan. Ecological Research 22: 331–341.

Nagamitsu, T., H. Yamagishi, T. Kenta, N. Inari, and E. Kato. 2010. Competitive effects of the exotic *Bombus terrestris* on native bumble bees revealed by a field removal experiment. Population Ecology 52: 123–136.

Nakamura, Y. 2014. Differences in pollen resource usage and foraging periods between the exotic bumblebee *Bombus terrestris* and the native *B. pseudobaicalensis* and *B. hypocrita sapporoensis* in Hokkaido, Japan. Eurasian Journal of Forest Research 17: 1–10.

Neumayer, J. 2006. Influence of honey-bees on nectar supply and native flower visitors. Entomologica Austriaca 13: 7-14.

Nielsen, A., J. Dauber, W. E. Kunin, E. Lamborn, B. Jauker, M. Moora, S. G. Potts, T. Reitan, S. Roberts, V. Sober, J. Settele, I. Steffan-Dewenter, J. C. Stout, T. Tscheulin, M. Vaitis, D. Vivarelli, J. C. Biesmeijer, and T. Petanidou. 2012. Pollinator community responses to the spatial population structure of wild plants: a pan-European approach. Basic and Applied Ecology 13: 489–499.

Nishikawa, Y., and T. Shimamura. 2016. Effects of alien invasion by *Bombus terrestris* L. (Apidae) on the visitation patterns of native bumblebees in coastal plants in northern Japan. Journal of Insect Conservation 20: 71–84.

Niwa, S., H. Iwano, S. Asada, M. Matsuura, and K. Goka. 2004. A microsporidian pathogen isolated from a colony of the European bumblebee, *Bombus terrestris*, and infectivity on Japanese bumblebee. Japanese Journal of Applied Entomology and Zoology 48: 60–64.

Ott, D., P. Hühn, and R. Classen-Bockhoff. 2016. *Salvia apiana*—a carpenter bee flower? Flora-Morphology, Distribution, Functional Ecology of Plants 221: 82–91.

Otterstatter, M. C., and J. D. Thomson. 2008. Does pathogen spillover from commercially reared bumble bees threaten wild pollinators? PLoS ONE 3: e2771.

Paini, D. R., and J. D. Roberts. 2005. Commercial honey bees (*Apis mellifera*) reduce the fecundity of an Australian native bee (*Hylaeus alcyoneus*). Biological Conservation 123: 103–112.

Paini, D. R., M. R. Williams, and J. D. Roberts. 2005. No short-term impact of honey bees on the reproductive success of an Australian native bee. Apidologie 36: 613–621.

Pedro, S. de M., and J. de Camargo. 1991. Interactions on floral resources between the Africanized honey bee *Apis mellifera* L. and the native bee community (Hymenoptera: Apoidea) in a natural “cerrado” ecosystem in southeast Brazil. Apidologie 22: 397–415.

Peng, W., J. Li, H. Boncristiani, J. P. Strange, M. Hamilton, and Y. Chen. 2011. Host range expansion of honey bee Black Queen Cell Virus in the bumble bee, *Bombus huntii*. Apidologie 42: 650–658.

Pick, R. A., and C. Schlindwein. 2011. Pollen partitioning of three species of Convolvulaceae among oligolectic bees in the Caatinga of Brazil. Plant Systematics and Evolution 293: 147–159.

Pinkus-Rendon, M. A., V. Parra-Tabla, and V. Melendez-Ramirez. 2005. Floral resource use and interactions between *Apis mellifera* and native bees in cucurbit crops in Yucatan, Mexico. Canadian Entomologist 137: 441–449.

Pleasants, J. M. 1981. Bumblebee response to variation in nectar availability. Ecology 62: 1648–1661.

Plischuk, S., and C. E. Lange. 2009. Invasive *Bombus terrestris* (Hymenoptera: Apidae) parasitized by a flagellate (Euglenozoa: Kinetoplastea) and a neogregarine (Apicomplexa: Neogregarinorida). Journal of Invertebrate Pathology 102: 261–263.

Plischuk, S., R. Martin-Hernandez, L. Prieto, M. Lucia, C. Botias, A. Meana, A. H. Abrahamovich, C. Lange, and M. Higes. 2009. South American native bumblebees (Hymenoptera: Apidae) infected by *Nosema ceranae* (Microsporidia), an emerging pathogen of honeybees (*Apis mellifera*). Environmental Microbiology Reports 1: 131–135.

Ravoet, J., L. De Smet, I. Meeus, G. Smagghe, T. Wenseleers, and D. C. de Graaf. 2014. Widespread occurrence of honey bee pathogens in solitary bees. Journal of Invertebrate Pathology 122: 55–58.

Richardson, M. L., C. P. Keathley, and C. L. Peterson. 2016. Breeding system of the critically endangered Lakela’s Mint and influence of plant height on pollinators and seed output. Population Ecology 58: 277–284.

Rogers, S. R., P. Cajamarca, D. R. Tarpy, and H. J. Burrack. 2013. Honey bees and bumble bees respond differently to inter- and intra-specific encounters. Apidologie 44: 621–629.

Roubik, D. W. 1978. Competitive interactions between Neotropical pollinators and Africanized honey bees. Science 201: 1030–1032.

Roubik, D. W. 1980. Foraging behavior of competing Africanized honeybees and stingless bees. Ecology 61: 836–845.

Roubik, D. W. 1983. Experimental community studies: time-series tests of competition between African and Neotropical bees. Ecology 64: 971–978.

Roubik, D. W., J. E. Moreno, C. Vergara, and D. Wittmann. 1986. Sporadic food competition with the African honey bee: projected impact on Neotropical social bees. Journal of Tropical Ecology 2: 97–111.

Roubik, D. W., and R. Villanueva-Gutierrez. 2009. Invasive Africanized honey bee impact on native solitary bees: a pollen resource and trap nest analysis. Biological Journal of the Linnean Society 98: 152–160.

Roubik, D. W., and H. Wolda. 2001. Do competing honey bees matter? Dynamics and abundance of native bees before and after honey bee invasion. Population Ecology 43: 53–62.

Sanguinetti, A., and R. B. Singer. 2014. Invasive bees promote high reproductive success in Andean orchids. Biological Conservation 175: 10–20.

Schaffer, W. M., D. B. Jensen, D. E. Hobbs, J. Gurevitch, J. R. Todd, and M. V. Schaffer. 1979. Competition, foraging energetics, and the cost of sociality in three species of bees. Ecology 60: 976–987.

Schaffer, W. M., D. W. Zeh, S. L. Buchmann, S. Kleinhans, M. V. Schaffer, and J. Antrim. 1983. Competition for nectar between introduced honey bees and native North-American bees and ants. Ecology 64: 564–577.

Semida, F., and S. Elbanna. 2006. Impact of introduced honey bees on native bees at St. Katherine Protectorate, South Sinai, Egypt. International Journal of Agriculture and Biology 8: 191-194.

Shavit, O., A. Dafni, and G. Ne’eman. 2009. Competition between honeybees (*Apis mellifera*) and native solitary bees in the Mediterranean region of Israel—implications for conservation. Israel Journal of Plant Sciences 57: 171–183.

Simpson, S. R., C. L. Gross, and L. X. Silberbauer. 2005. Broom and honeybees in Australia: an alien liaison. Plant Biology 7: 541–548.

Singh, R., A. L. Levitt, E. G. Rajotte, E. C. Holmes, N. Ostiguy, D. vanEngelsdorp, W. I. Lipkin, C. W. dePamphilis, A. L. Toth, and D. L. Cox-Foster. 2010. RNA viruses in hymenopteran pollinators: evidence of inter-taxa virus transmission via pollen and potential impact on non-*Apis* hymenopteran species. PLoS ONE 5: e14357.

Smith-Ramírez, C., R. Ramos-Jiliberto, F. S. Valdovinos, P. Martínez, J. A. Castillo, and J. J. Armesto. 2014. Decadal trends in the pollinator assemblage of *Eucryphia cordifolia* in Chilean rainforests. Oecologia 176: 157–169.

Steffan-Dewenter, I., and T. Tscharntke. 2000. Resource overlap and possible competition between honey bees and wild bees in central Europe. Oecologia 122: 288–296.

Stout, J. C., A. R. Kells, and D. Goulson. 2002. Pollination of the invasive exotic shrub *Lupinus arboreus* (Fabaceae) by introduced bees in Tasmania. Biological Conservation 106: 425–434.

Sugden, E. A., and G. H. Pyke. 1991. Effects of honey-bees on colonies of *Exoneura asimillima*, an Australian native bee. Australian Journal of Ecology 16: 171–181.

Sun, S.-G., S.-Q. Huang, and Y.-H. Guo. 2013a. Pollinator shift to managed honeybees enhances reproductive output in a bumblebee-pollinated plant. Plant Systematics and Evolution 299: 139–150.

Sun, S.-G., B. R. Montgomery, and B. Li. 2013b. Contrasting effects of plant invasion on pollination of two native species with similar morphologies. Biological Invasions 15: 2165–2177.

Szabo, N. D., S. R. Colla, D. L. Wagner, L. F. Gall, and J. T. Kerr. 2012. Do pathogen spillover, pesticide use, or habitat loss explain recent North American bumblebee declines? Conservation Letters 5: 232–239.

Tatsuno, M., and N. Osawa. 2016. Flower visitation patterns of the coexisting honey bees *Apis cerana japonica* and *Apis mellifera* (Hymenoptera: Apidae). Entomological Science 19: 255–267.

Taylor, G., and R. Whelan. 1988. Can honeybees pollinate *Grevillea*? Australian Zoologist 24: 193–196.

Tepedino, V. J., D. G. Alston, B. A. Bradley, T. R. Toler, and T. L. Griswold. 2007. Orchard pollination in Capitol Reef National Park, Utah, USA. Honey bees or native bees? Biodiversity and Conservation 16: 3083–3094.

Thomson, D. 2004. Competitive interactions between the invasive European honey bee and native bumble bees. Ecology 85: 458–470.

Thomson, D. M. 2006. Detecting the effects of introduced species: a case study of competition between *Apis* and *Bombus*. Oikos 114: 407–418.

Thomson, D. M. 2016. Local bumble bee decline linked to recovery of honey bees, drought effects on floral resources. Ecology Letters 19: 1247–1255.

Torne-Noguera, A., A. Rodrigo, S. Osorio, and J. Bosch. 2016. Collateral effects of beekeeping: impacts on pollen-nectar resources and wild bee communities. Basic and Applied Ecology 17: 199–209.

Walther-Hellwig, K., G. Fokul, R. Frankl, R. Buechler, K. Ekschmitt, and V. Wolters. 2006. Increased density of honeybee colonies affects foraging bumblebees. Apidologie 37: 517–532.

Whitehorn, P. R., M. C. Tinsley, M. J. F. Brown, and D. Goulson. 2013. Investigating the impact of deploying commercial *Bombus terrestris* for crop pollination on pathogen dynamics in wild bumble bees. Journal of Apicultural Research 52: 149–157.

Wilms, W., and B. Wiechers. 1997. Floral resource partitioning between native *Melipona* bees and the introduced Africanized honey bee in the Brazilian Atlantic rain forest. Apidologie 28: 339–355.

Woods, T. M., J. L. Jonas, and C. J. Ferguson. 2012. The invasive *Lespedeza cuneata* attracts more insect pollinators than native congeners in tallgrass prairie with variable impacts. Biological Invasions 14: 1045–1059.

Xia, J., S. G. Sun, and Y. H. Guo. 2007. Honeybees enhance reproduction without affecting the outcrossing rate in endemic *Pedicularis densispica* (Orobanchaceae). Plant Biology 9: 713–719.
